# Nucleation in confinement generates long-range repulsion between rough calcite surfaces

**DOI:** 10.1038/s41598-019-45163-6

**Published:** 2019-06-20

**Authors:** Joanna Dziadkowiec, Bahareh Zareeipolgardani, Dag Kristian Dysthe, Anja Røyne

**Affiliations:** 1 The NJORD Centre, Physics of Geological Processes (PGP), Department of Physics, University of Oslo, Oslo, 0371 Norway; 2 Institut Lumière Matière, Université de Lyon, Université Claude Bernard Lyon 1, CNRS UMR 5586, Campus de la Doua, F-69622 Villeurbanne, cedex France

**Keywords:** Mineralogy, Chemical physics, Surfaces, interfaces and thin films, Structural properties, Structure of solids and liquids

## Abstract

Fluid-induced alteration of rocks and mineral-based materials often starts at confined mineral interfaces where nm-thick water films can persist even at high overburden pressures and at low vapor pressures. These films enable transport of reactants and affect forces acting between mineral surfaces. However, the feedback between the surface forces and reactivity of confined solids is not fully understood. We used the surface forces apparatus (SFA) to follow surface reactivity in confinement and measure nm-range forces between two rough calcite surfaces in NaCl, CaCl_2_, or MgCl_2_ solutions with ionic strength of 0.01, 0.1 or 1 M. We observed long-range repulsion that could not be explained by changes in calcite surface roughness, surface damage, or by electrostatic or hydration repulsion, but was correlated with precipitation events which started at µm-thick separations. We observed a submicron-sized precipitate that formed in the confined solution. This liquid-like viscous precipitate did not undergo any spontaneous ripening into larger crystals, which suggested that confinement prevented its dehydration. Nucleation was significantly postponed in the presence of Mg^2+^. The long-range repulsion generated by nucleation between confined mineral surfaces can have a crucial influence on evolution of the microstructure and therefore the macroscopic strength of rocks and materials.

## Introduction

Fluid-driven mineral reactions in nm- to µm-wide confined spaces can significantly differ from bulk processes as small fluid volumes, slow diffusion and limited advection may promote mineral growth^1^. Reactive mineral contacts at grain boundaries and fracture tips frequently govern the macroscopic mechanical strength of rocks and building materials^2,3^. However, it is not clear what is the relative importance of crystallization and interfacial forces in determining the strength of individual solid-solid contacts. In geological environments, nm-range surface forces are relevant down to several km depth in the subsurface. In these regions, MPa-range positive disjoining pressures^4^ (or repulsive forces) can sustain the overburden pressure, and thus nm-thin water films can be maintained between contacting mineral surfaces^5^. Recent experimental^6,7^ and modelling studies^8,9^ of confined single crystal precipitation suggest that there is a strong link between confined mineral growth and the presence of repulsive surface forces that control the thickness of the water films separating the surfaces. The feedback between surface forces and confined mineral growth needs to be further examined.

Calcite is a major mineral resource and biomineral. It is also a common accessory mineral in the Earth’s crust and builds vast chalk and limestone sediments. These carbonate rocks are porous and prone to chemical compaction because of the relatively high reactivity of calcite in contact with percolating fluids^10^. The reactivity of calcite in the confined interfacial regions may significantly contribute to either rock consolidation by cementation^11,12^ or weakening by brittle and plastic deformation^13,14^. Although recent studies related to carbonate-fluid interactions have suggested that surface forces may influence the mechanical strength of carbonate rocks^12,15–18^, direct measurements of the forces between calcite surfaces in aqueous solutions varying in ionic strength and composition are limited^16,19–22^.

Salinity has a pronounced effect on nm-range forces between two calcite surfaces. Strong repulsive hydration forces due to hydration of the highly hydrophilic calcite surface have been recently measured both in water and in electrolyte solutions^16,20^ and found to significantly exceed the electrical double layer repulsion. The onset and magnitude of the hydration forces have largely depended on the electrolyte concentration, with smaller onsets at higher concentrations^20^. The collapse of surface hydration layers at high ionic strengths (>0.1 M NaCl) and electrostatic attraction due to ion correlation may be the two dominant mechanisms that facilitate adhesion between calcite surfaces, as suggested by Javadi and Røyne^22^. Adhesive forces between two calcite surfaces have also been measured at strongly alkaline conditions (pH = 12, 0.12 M), pointing to weaker repulsion at low calcite zeta potentials^19^.

Salinity also influences calcite reactivity. The salinity of pore waters that saturate sedimentary rocks can vary within 5 orders of magnitude, reaching as high as 0.3 kg/L (~5 M NaCl) of dissolved solids^23^. Mixing, migration of these waters, and anthropogenic injection of fluids into carbonate rocks can lead to temporary disequilibrium conditions and activation of growth and dissolution processes. Calcite solubility and growth kinetics in salt solutions are mainly affected by changes in ionic strength, ion hydration, ion pairing, and the common ion effect^24,25^. As the solution ionic strength increases, the activity of species that build the solid phase decreases in the solution, causing a higher solubility of calcite^26^. The dissolution rate of calcite has been found to increase at higher ionic strengths (>1 mM), owing to the ion-specific changes in Ca^2+^ solvation and the resulting disruption of calcite surface hydration layers^27,28^. Background ions also have a profound impact on CaCO_3_ nucleation, since they affect the dehydration energy of emerging nucleation clusters and therefore lead to significant differences in the critical supersaturation required for nucleation^25^. Certain ions that can be incorporated into calcite lattice (e.g. Mg^2+^), will additionally modify the calcite solubility due to the impurity effect^29^.

Spatial confinement can have a manifold effect on calcite reactivity. Ion depletion and reduced ion mobility in pores make nucleation events less probable^30,31^, which increases induction times for crystallization. Single, µm-sized crystals grown in confinement display diffusion-limited rim topographies^7,32^. At the nanoscale, confinement effects may be even more pronounced: If the pore dimensions are smaller than the critical nuclei size, the surface free energy barrier may prevent nucleation altogether^33^. Nanoporous materials may selectively control the growth of different CaCO_3_ polymorphs^34^, and pore size-related changes in ion distribution near charged surfaces may promote growth of otherwise unstable phases^35^. Interestingly, Stephens, *et al*.^36^ have recently observed that even µm-range confinement can slow down or prevent the transformation of amorphous calcium carbonate (ACC) into the more stable CaCO_3_ polymorphs. The authors suggested that despite the lower surface free energy of ACC with respect to calcite, ACC stabilization could not have been of thermodynamic origin since the bulk free energy gain on recrystallization into calcite dominated for surface separations larger than a few nm. They therefore attributed the stabilization of ACC to kinetic effects related to restricted ion transport in the confined solution^36^.

It is not clear how changes in salinity affect interactions between confined calcite surfaces. On one hand, attractive, short range forces between calcite surfaces should dominate in concentrated electrolyte solutions, leading to strengthening of grain contacts^22^. On the other hand, calcite surfaces become more soluble and reactive in high salinity solutions, which could make the interfaces weaker. The surface reactivity of confined calcite interfaces can lead to transport-dependent recrystallization processes and major increase in surface roughness^21^. Roughness and crystal growth may in turn give rise to very strong repulsive forces linked with the force of crystallization^3^ and nanoscale asperity deformation^37,38^. It is also not clear in which conditions mineral growth in confinement can lead to contact cementation^7^.

Measurements of surface forces and reactivity of confined mineral interfaces remains a challenge since few methods are able to follow both the forces and topographical evolution *in situ* with sufficient resolution. In this work, we used the surface forces apparatus (SFA)^39,40^ coupled with multiple beam interferometry (MBI)^41–44^ to measure both the nm-range forces between two rough, polycrystalline calcite surfaces and their surface reactivity in confinement, the latter with µm-scale resolution. We performed the measurements in NaCl, CaCl_2_ and MgCl_2_ electrolyte solutions with ionic strength ranging from 10 mM to 1 M. The geometry of two contacting surfaces in our SFA experiment resembles an open slit pore with nm to sub-µm distance between the two opposing walls in the contact area with a typical radius of 50–100 µm. In contrast to a standard Atomic Force Microscopy (AFM) experiment with nm-sized contact areas, such a large contact area significantly affects the transport of ionic species and thus surface reactivity^21^. As such, our sample setup is relevant for confined interfaces not only in geological environments but also in granular, mineral-based materials.

## Results and Discussion

**Figure 1:**
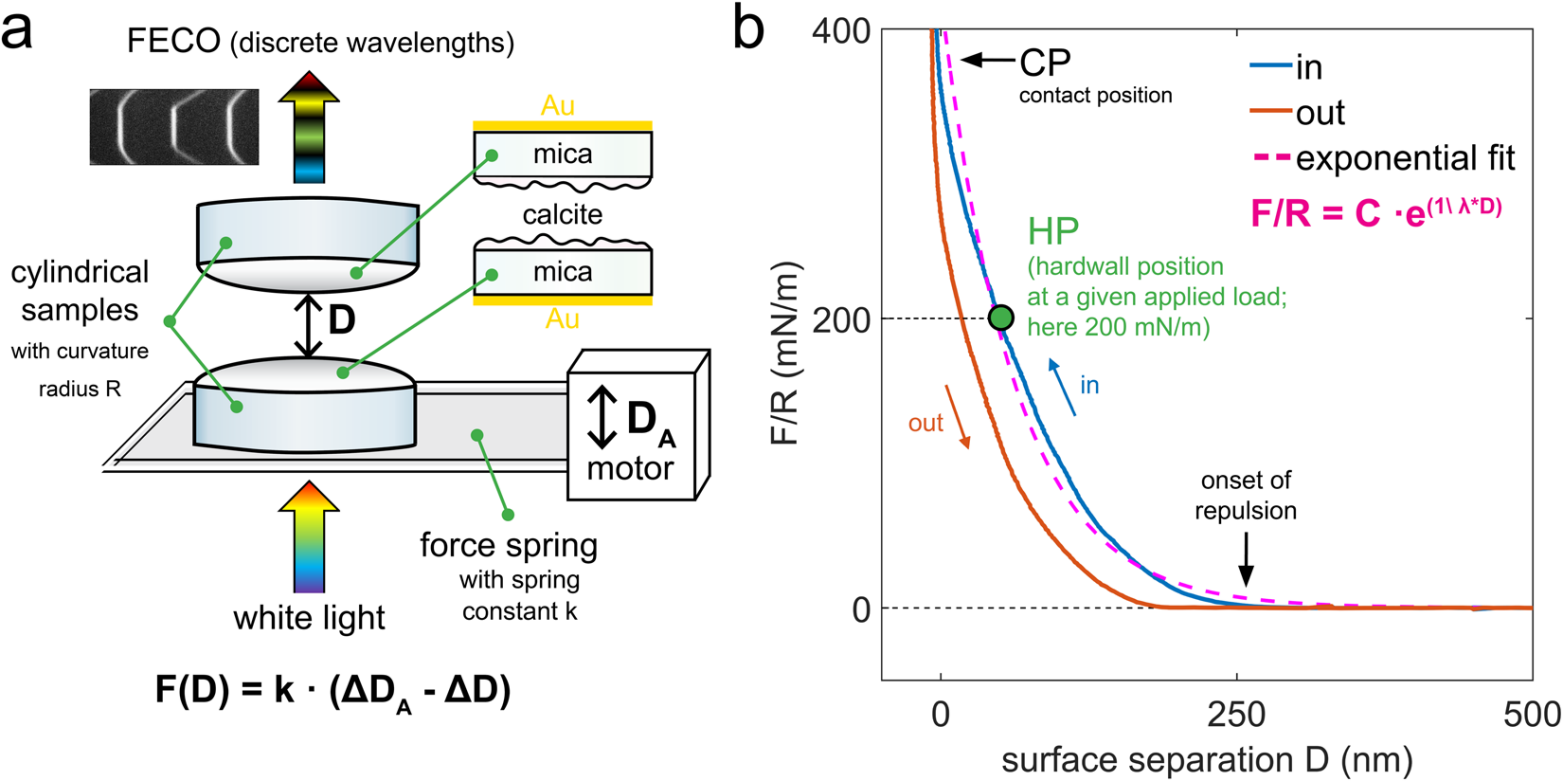
(**a**) Schematic representation of the SFA setup with two calcite surfaces glued to two crossed cylindrical disks with radius of curvature R. Surfaces are mounted on a force measuring spring, which is driven up and down at a constant velocity by a distance D_A_. The actual distance between the surfaces D_M_ is measured by optical MBI technique: fringes of equal chromatic order (FECO) form after passing white light through semi-reflective samples with nm-thick Au mirrors. Mica is used as a support to deposit calcite and Au films; (**b**) Representative SFA force (F) measurement showing F normalized with R as a function of surface separation D on approach (in) and on retraction (out). Important parameters were marked on the plot. CP is defined as the distance at which the separation between the surfaces does not decrease significantly despite the continued loading. Exponential fit to the force-distance curve on approach is used to determine magnitude and range of repulsion by using exponential decay length λ; C is a fitting coefficient.

We used the SFA to measure forces (*F*) as a function of surface separation (*D*) between rough and polycrystalline calcite surfaces in three calcite-saturated electrolyte solutions: NaCl, CaCl_2_ and MgCl_2_ with ionic strengths (*IS*) of 0.01, 0.1 and 1 M. During the SFA experiments, we performed repeated loading-unloading cycles, in which a bottom calcite surface (mounted on a force measuring spring) was approached towards and retracted from a top calcite surface at a constant velocity (ranging from 1 to 10 nm/s). In the SFA, the distance between surfaces is measured with an optical multiple beam interferometry (MBI) technique: the wavelength positions of a set of fringes of equal chromatic order (FECO; that result from light transmission through two semi-transparent samples) correspond to a given separation between surfaces. Positions of the FECO fringes are also sensitive to refractive indices of layered samples. The SFA setup and the most important parameters of measured force curves are shown in Fig. 1. The details of SFA and MBI techniques and preparation of calcite samples for the SFA have been previously described^21,40–42,45^.

We observed a clear and reproducible pattern of events during our SFA experiments: (1) Forces between two calcite surfaces were initially monotonically repulsive, with no resolved attraction or adhesion in any of the solutions, even for the smoothest calcite surfaces; (2) calcite surfaces initially dissolved in contact with all solutions, and in most of the experiments, these dissolution periods were followed by major precipitation events; (3) Immediately before the precipitation events, we measured a significant increase in the magnitude and range of repulsive forces; (4) During these events, we could identify distinct *precipitation fronts* (PF) with the growing precipitate spreading into the contact regions between calcite surfaces; and (5) After the passage of the *precipitation fronts*, the magnitude and onset (taken as the distance at which the force becomes of measurable magnitude; Fig. 1b) of the repulsive forces substantially increased, to the extent that it could not be explained by roughening or damage of the calcite samples. In the following sections we first discuss the origin of the moderately repulsive forces before PFs, then we characterize PFs, and last, we discuss the long-range repulsive forces measured after PFs.

### Calcite surfaces

**Figure 2:**
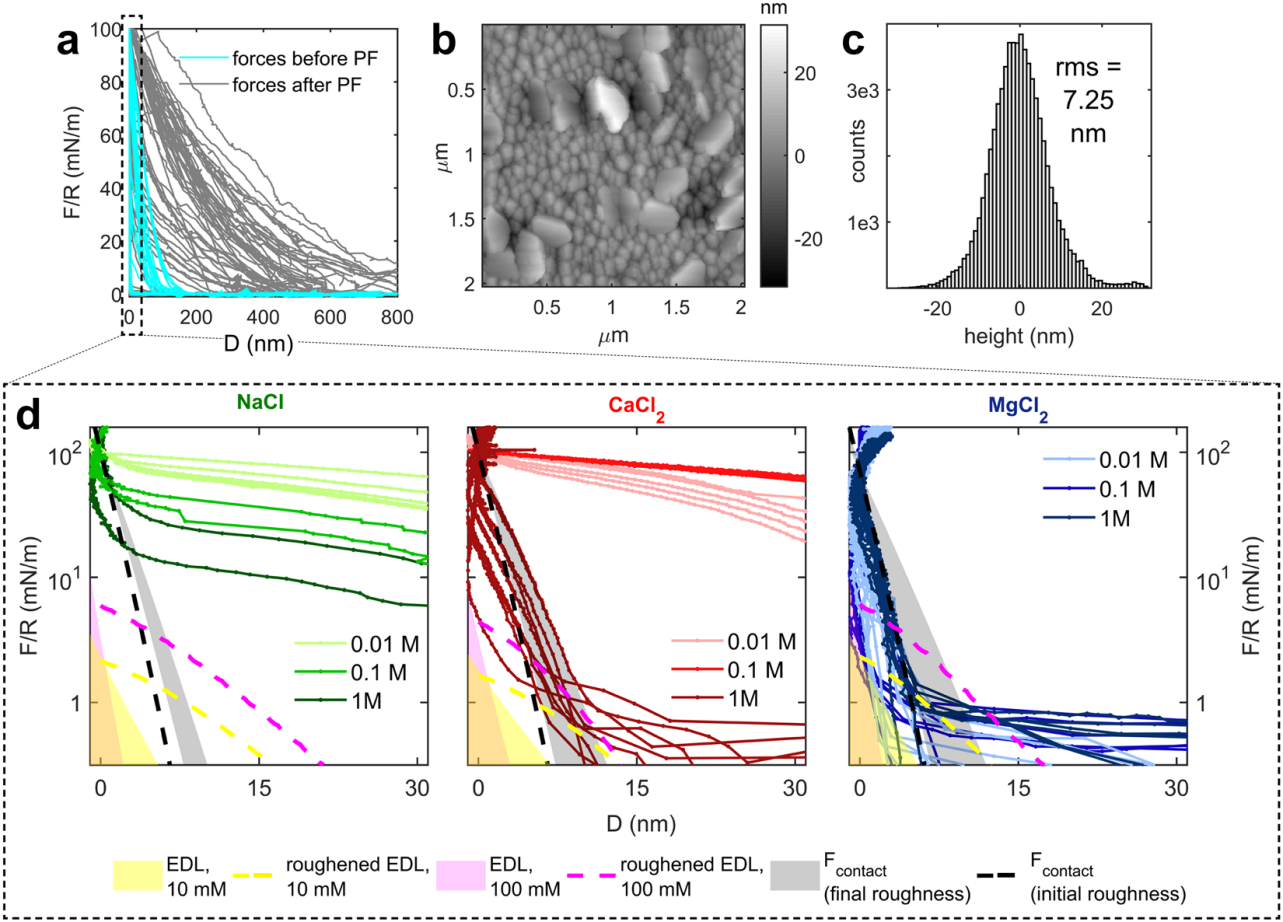
SFA force measurements between two rough polycrystalline set 2 calcite surfaces. (**a**) Summary of forces (F/R; normalized with radius of curvature R) measured as a function of surface separation (D) before and after passage of precipitation fronts (PF). Note much smaller range and magnitude of the repulsion measured before PFs; (**b**) Representative AFM height map for the used calcite surfaces measured before the SFA experiments; (**c**) Histogram of surface heights corresponding to B; (**d**) Measured force curves (whole drawn lines) between two calcite surfaces (set 2) before PF events (cyan force curves in subplot A) in NaCl, CaCl_2_ and MgCl_2_ electrolyte solutions with IS = 0.01 to 1 M, along with the modelled electrical double layer (EDL) repulsion and two roughness contributions (F_contact_ and roughened EDL).

In line with previous findings^21,46^, X-ray Diffraction (XRD) indicated that all the CaCO_3_ films prepared by Atomic Layer Deposition (ALD) were composed of calcite (Fig. S4). Two sets of ALD calcite surfaces were used for two sets of SFA experiments (i.e. set 1 corresponds to set 1 surfaces and set 1 experiments). Although the ALD deposition parameters were kept constant (Table S3), these films differed in morphology and initial roughness (Figs S5–S8) due to high sensitivity of the ~8 h deposition process to the deposition parameters and substrate characteristics^46^. Set 1 surfaces were composed of small crystals (50–100 nm), with a relatively high amount of much larger (~1 µm), polycrystalline aggregates particles on the surfaces (Figs S5, S7). The root-mean-square (rms) roughness of the set 1 films varied by almost 2 orders of magnitude due to the random distribution of the large aggregates (Figs S8, S5C). Set 2 surfaces were more homogenous with larger, platy crystals (>200 nm) and continuous coverage of smaller crystals (50–100 nm; Figs 2b,c, S6–7), and an initial average rms value of 4.3 ± 0.8 nm (as measured in 3 positions on a sample, scan size 15 × 15 µm^2^; Fig. S8). Despite using calcite-saturated electrolyte solutions in our experiments, we observed a minor initial dissolution of all the calcite films. This was mainly related to: (a) disequilibrium morphology of calcite crystals grown by ALD from vapor phase, with the possible presence of high-energy crystal faces, as reported previously^21,46^; (b) large roughness of the substrate composed of nm-sized crystals with large surface to volume ratio^47^; and (c) to changes in partial pressure of CO_2_ (pCO_2_) during experiments due to minor equilibration of electrolyte solutions in the sealed SFA chamber with the atmospheric CO_2_ (pCO_2_ = 10^−3.5^ atm; Table S2). By performing a control experiment with a solution in equilibrium with atmospheric pCO_2_ (Fig. S3, section S4), and by considering the changes in Gibbs free energy (ΔG) in our system, we estimated that a) and b) are the two main driving forces for dissolution of rough ALD calcite films (Fig. S1; see details of calculations in section S3 of the supplementary information). The films can also undergo pressure-induced dissolution due to the repeatedly applied load during SFA measurements. Nevertheless, our calcite films remained intact and continuous throughout the SFA experiments, even when 1 M *IS* solutions were used. We additionally measured the evolution of surface roughness for unconfined ALD calcite surfaces (set 3) using the AFM in 0.01 and 1 M *IS* NaCl, CaCl_2_ and MgCl_2_ solutions, presaturated with calcite (Figs S9–10). We observed minor changes in surface roughness within several hours (Δ*rms* < 3 nm). Only the most concentrated (1 M) NaCl solutions induced substantial dissolution of the unconfined calcite films in the AFM, with µm-sized dissolution pits developing on the surfaces within the first 4 h (Fig. S10).

### Origin of the repulsive forces before the precipitation fronts

In each SFA experiment, we measured forces as a function of surface separation for the same µm-sized contact for 2 days (set 1) or 1 day (set 2). In this section, we only discuss the moderately repulsive forces measured in the initial stages of the experiments, before PFs (Fig. 2a,d). The forces measured before PFs were monotonically repulsive and we could not resolve any attractive or adhesive forces, even for the solutions with high ionic strength. We semi-quantified the magnitude and onset of the repulsion using the decay length λ of the exponential fit to the force-distance curves measured on approach^21,38,48^ (Fig. 1b).

Figure 2d shows all force curves obtained on approach for set 2 surfaces, measured before PFs in NaCl, CaCl_2_ and MgCl_2_ solutions. Set 2 surfaces were smooth enough that small applied loads were sufficient to reach the contact position (CP; defined as the distance at which the separation between the surfaces does not decrease significantly despite continued loading; Fig. 1b). Flattening of the FECO fringes^45^ observed at CP additionally indicated that surfaces were in a very close proximity: the minimum separations between surfaces were initially <10 nm over the whole nominal contact areas (~100 µm) for most of the set 2 experiments (Figs 2d; S13). For these experiments, we observed major differences in the range of repulsion in different solutions. We measured relatively long-range repulsion (with onsets at >100 nm and 16 nm < λ < 65 nm) in NaCl and in 0.01 and 0.1 M CaCl_2_ solutions (Fig. 2d). Shorter-range repulsion (with onsets <15 nm and 1 nm < λ < 6 nm) was measured in MgCl_2_ and 1 M CaCl_2_ (Fig. 2d).

Set 1 surfaces were much rougher than set 2 surfaces and large, µm-sized asperities (Fig. S5C) prevented the surfaces from reaching CP. These asperities gave rise to comparable decay lengths for the set 1 and set 2 surfaces (set 1: 6 nm < λ < 35 nm; set 2: 1 nm < λ < 65 nm), because they acted as discrete hard walls at large separations (<1 µm; Fig. S13), and very high loads had to be applied to move the surfaces further in (as these large asperities plastically deformed); nm-range separations, at which surface forces operate, were thus only accessible for the highest asperities in the contact region. This explains why we did not resolve any major differences related to ionic strength or solution composition for the rougher set 1 surfaces.

As we observed major differences between decay lengths of repulsive force curves measured for the smoother set 2 surfaces in different electrolyte solutions (long-range repulsion in in NaCl and in 0.01 and 0.1 M CaCl_2_ and short-range repulsion in MgCl_2_ and 1 M CaCl_2_), we modelled which type of forces could explain the variation in the range of repulsion (Fig. 2d). We considered possible contributions of: Van der Waals (VdW) forces, hydration forces, roughness and electric double layer (EDL) forces. As explained in the Supplementary Information (section S9), we neglected the attractive VdW forces and repulsive hydration forces. For the remaining two repulsive terms, we treat the effect of roughness explicitly and show that neither roughness nor EDL forces were sufficient to explain the measured variation in the range and magnitude of the repulsion. We suggest that the long-range repulsion was related to nucleation in the solution confined between the surfaces, even before the observable PFs, as explained below.

The magnitude and range of EDL repulsive forces between similar surfaces are related to the surface charge and ionic strength of the solution. A precise determination of the EDL contribution for two calcite surfaces is challenging because of the large variation in reported calcite zeta potentials and their sensitivity to pCO_2_ and solution composition^49–53^, as well as few reported values for the calcite surface charge regulation parameters^20^, which cannot be measured using rough and reactive calcite surfaces. Therefore, we chose to consider the possible range of EDL forces corresponding to the absolute zeta potential values of 5 to 30 mV, typically reported for calcite at pH ~8–9^53^. The EDL force contribution was calculated using a linearized Poisson-Boltzmann equation and calcite charge regulation parameter estimated by Diao and Espinosa-Marzal^20^. Details of the calculations are outlined in the Supplementary Information (section S9). The calculated Debye length of our electrolyte solutions varied between 3.0–4.3 nm for the 0.01 M electrolytes and 1.0–1.4 nm for the 0.1 M electrolytes (as calculated including Ca^2+^, CO_3_^2−^, HCO_3_^−^ species due to calcite dissolution upon pre-saturation, using PhreeqC). At 1 M, DLVO theory breaks down and EDL forces should be negligible due to strong ionic screening^54^. As such, the maximum range of the theoretically calculated EDL is ~15–30 nm for smooth calcite surfaces in our most dilute 2:1 electrolytes and at the highest surface charge (30 mV; Fig. S11).

Surface roughness contributes to the measured repulsion in two ways. First, roughness produces repulsive mechanical effects due to plastic and elastic deformation of surface asperities on loading, the magnitude of which generally increases exponentially (for surfaces with random distribution of surface heights such as our set 2 calcite surfaces; Fig. 2c) with decreasing surface separation^37,38^. The onset of this repulsion is related to the distance at which the first large asperities come into contact, roughly at distances smaller than 3 times the rms roughness of the surfaces^38^. Second, roughness smears out any distance-dependent interaction potential due to variation of surface heights across the nominal contact area^38,55^. Due to disruption of ion layering near the surface and possible roughness-related variations of surface charge, these roughness effects may extend over the full width of the EDL^56^.

To account for these two roughness contributions, we used the model proposed by Parsons, *et al*.^38^. We estimated the first roughness contribution due to elastic or plastic asperity deformation from *Eq*. 16 in Parsons, *et al*.^38^ (F_contact_; Fig. 2d) This contribution is based on rms roughness (measured with AFM for the ALD surfaces before (F_contact_ initial) and after (F_contact_ final) the SFA experiment, at three random positions for each surface; scan size 15 × 15 µm^2^), average asperity radius (approximated from the AFM maps by measuring radii of areas above a height threshold of 70%), and the Young’s modulus and Poisson ratio of calcite. The second roughness contribution due to variation of surface heights across the nominal contact area was calculated using *Eq*. 7 in Parsons, *et al*.^38^ (roughened EDL; Fig. 2d) This contribution was modelled by averaging the theoretical EDL force (calculated with *Eq*. S2 for *ψ*_0_ = −30 mV) for smooth calcite surfaces over the distribution of surface heights measured with the AFM for each surface (scan size 15x15 µm^2^). The Derjaguin approximation was used to relate the calculated roughness-related interaction energy to the force acting between two cylindrical SFA samples (see *Eq*. 1 in Parsons, *et al*.^38^).

The results of force modelling (Fig. 2d) indicated that the EDL and roughness force contributions are not sufficient to explain the long-range repulsion measured in NaCl and in 0.01 and 0.1 M CaCl_2_ solutions: (1) EDL repulsive forces calculated for smooth calcite surfaces can be of measurable magnitude at separations <10 nm. The onset of EDL forces may be larger (separations >15 nm) if we consider roughness-averaged EDL forces (roughened EDL). However, even the roughened EDL cannot explain the measured long-range repulsion with onsets >100 nm (and 16 nm < λ < 65 nm). EDL forces may significantly contribute to the short-range repulsion measured in MgCl_2_ and 1 M CaCl_2_, however it is not possible to precisely distinguish it from the roughness F_contact_ contribution, which becomes of significant magnitude at comparable separations; (2) Roughness contribution due to asperity deformation (F_contact_) can explain the high-magnitude, short-range repulsion with onsets below 15 nm (and 1 nm < λ < 6 nm) measured for the experiments in MgCl_2_ and 1 M CaCl_2_ solutions. The magnitude and range of the experimentally measured repulsion in these experiments corresponds very well to the F_contact_ force that was calculated using the roughness parameters measured for the probed calcite surfaces with the AFM. Since the roughness of set 2 surfaces was homogeneous and comparable for all samples (Fig. S8C,D), repulsive forces due to surface roughness cannot explain the long-range repulsive forces measured in NaCl and 0.01 and 0.1 M CaCl_2_ solutions.

What is then the potential origin of the long-range repulsion that we measured before the observable PFs? If roughness was to explain the long-range repulsion in NaCl and 0.01 and 0.1 M CaCl_2_, then the rms roughness of these surfaces (according to *Eq. 16* in Parsons, *et al*.^38^), would need to be one order of magnitude higher than measured with the AFM (up to rms ~100 nm for 0.01 M NaCl). We did not measure such a high roughness for any of the calcite surfaces used in the experiments in which the long-range repulsive forces were present. Since, apart from AFM, we also investigated each sample with the Scanning Electron Microscopy (SEM; Figs S5–6), it is unlikely that we overlooked features on the surface that could give rise to such large roughness.

Possible sample damage during the experiment, such as large calcite particles (~0.1 µm) breaking off and becoming trapped between the surfaces, could potentially explain the long-range repulsion with onsets >100 nm. Although we do not observe any loose particles in the camera or any major irregularities in FECO fringes, the size of such particles could have been below the µm-range resolution of the FECO and our camera. However, with large particles trapped between the surfaces we would not observe a pronounced flattening of the surfaces (due to elastic deformation of the glue) at the contact position, as the pressure would be concentrated on the discrete asperity contacts that are much smaller than the nominal contact area in the SFA. The flattening was observed for all set 2 experiments before the PFs.

Ruling out changes in calcite surface roughness, EDL forces and surface damage as explanations for the initial presence of long-range and high-magnitude repulsive forces leaves us to consider the properties of the solution confined between two calcite surfaces. In the following, we will show that the fluid compositions with presence of long-range and high-magnitude repulsive forces are the compositions where we later observed distinct precipitation fronts. As such, the long-range repulsion measured before the PFs was likely related to nucleation of CaCO_3_ between the calcite surfaces.

### Precipitation fronts

**Figure 3:**
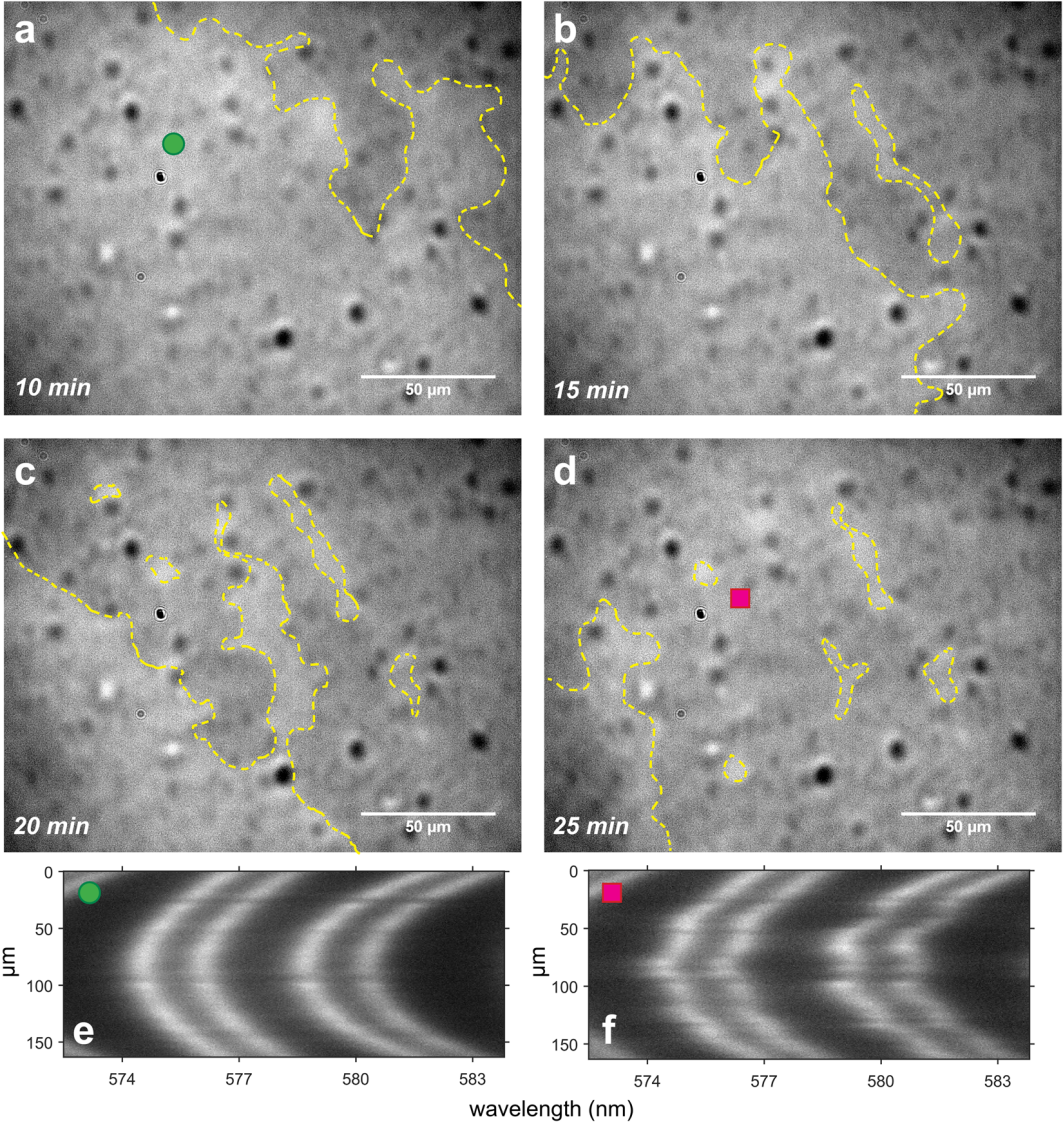
(**a**–**d**) Spreading of precipitation front (PF) between two calcite surfaces in the SFA, indicated by a darker color of the precipitate (set 1, 0.1 M NaCl experiment; supplementary movie M2); (**e**) FECO fringes before PF (surfaces out of contact); (**f**) FECO fringes after PF (surfaces out of contact). The center of the contact area established in the SFA (corresponding to the shown FECO fringes) is approximately indicated with a green or a pink symbol.

In almost all SFA experiments, we observed distinct *precipitation fronts* (PFs) passing through the imaged contact regions (Fig. 3). PFs were manifested by fingering growth of a darker region between the two calcite surfaces, spreading into the spherical contact area from outside of the contact with velocities ranging from ~10 to ~500 nm/s (Fig. 3a–d, Supplementary Movies M1–M14). The PFs could be identified in the camera by a change in intensity of the light (transmitted through two semi-transparent calcite surfaces in the SFA) from brighter to darker, which was most likely related to a change of the refractive index of the solution trapped between the surfaces (or to a change in separation between surfaces – but the irregular patterns formed by the spreading precipitate make this less likely). The PFs could be also identified from the changing position and shape of the FECO fringes^42,45^, which were gradually losing their resolution (became wider) and became very irregular (Fig. 3e,f). Whenever PFs reached the contact region, positions of FECO fringes shifted to wavelengths corresponding to larger separations (as determined from experiments in which surfaces were kept at a fixed separation under constant load during PF). The irregular shape of the FECO fringes could indicate changes in surface topography or uneven refractive index (and thus uneven density) of the solution confined between the surfaces. Since we did not observe any major changes in calcite surfaces topography after the SFA experiments (Fig. S8), the irregularity of the FECO fringes likely indicated variations in the density of the solution confined in the contact region. Based on these observations, we interpret these fronts to represent precipitation events.

**Figure 4:**
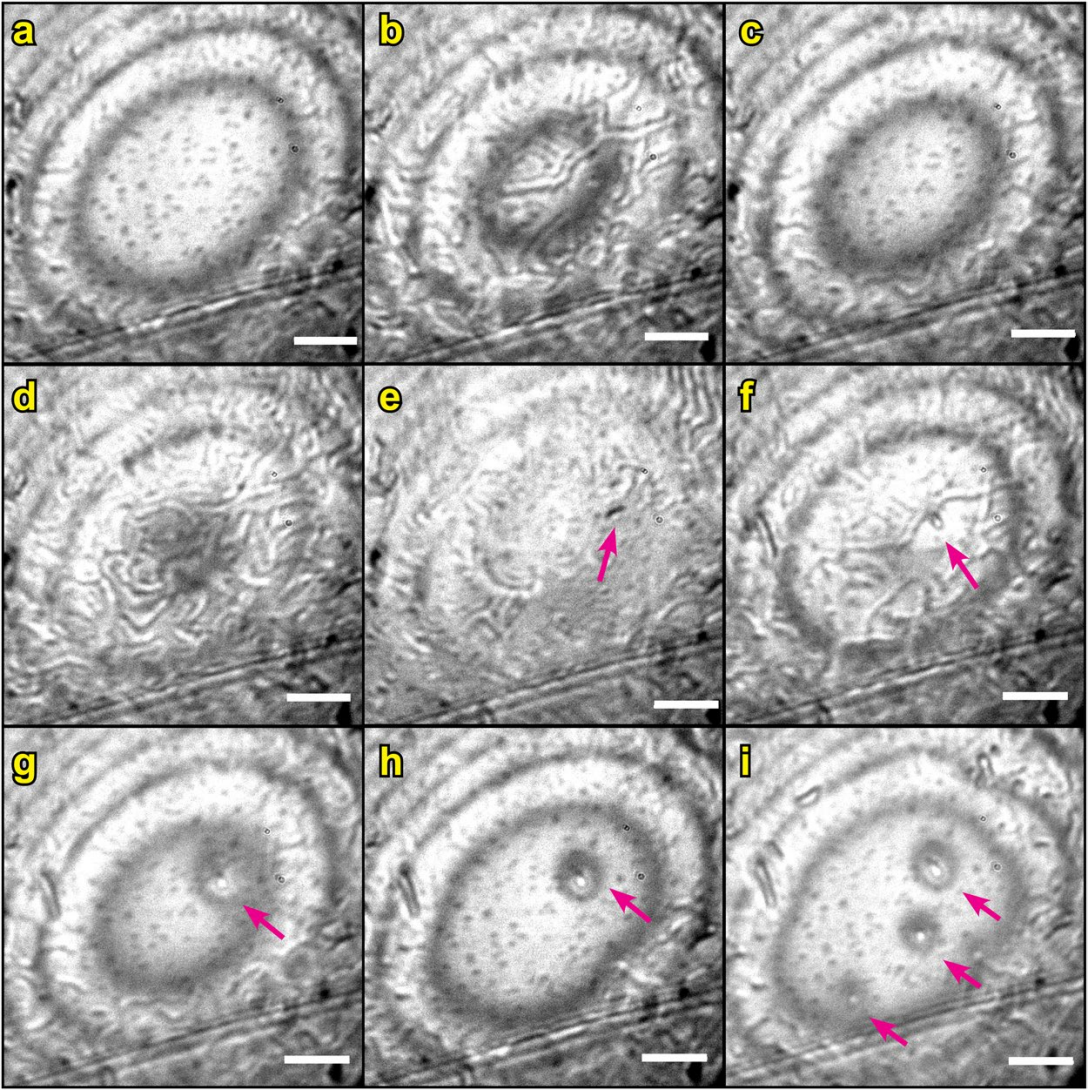
Contact region between two calcite surfaces in the SFA after PF. Newton rings (interference fringes) connect regions of the same surface separation. The bright central Newton ring indicates a contact region of the smallest separation. The larger the diameter of the central Newton ring, the larger the nominal contact area; scale bar is 50 µm. Precipitate is identified as irregular, twisted features in the images. (**a**,**c**) Precipitate is squeezed out of the contact region when the surfaces are approached manually at high loads. (**b**,**d**) Precipitate flows back into the contact region upon surface separation. (**e**–**i**) After several loading-unloading cycles, µm-sized crystals grow between the surfaces (indicated with arrows), first when the surfaces are out of contact. Images a to i are a sequence in time (see also the supplementary movie M16).

We had no possibility to directly identify the material precipitating between the calcite surfaces, but since we could not resolve any distinct particles or crystals, the precipitate was most likely composed of submicron particles. This is also supported by no observable loss in the FECO fringes intensity (Fig. 3f): micron-sized particles would scatter the light and make FECO discontinuous or dim. Given the chemical composition of the surfaces and solutions, it is unlikely that any mineral phase other than CaCO_3_ would precipitate. We did not observe any distinct precipitate after the SFA experiments with AFM or SEM (Figs S5–6), suggesting that the precipitate remained in the confined solution that was mixed into the bulk solution on disassembling the SFA surfaces. Observations in the camera, when repeatedly approaching and separating the surfaces, suggested that the new phase was a viscous liquid-like dispersion of the submicron precipitate particles, which could flow into and out of the contact region (Supplementary Movie M15, Fig. 4a–d).

We cannot unambiguously conclude if the precipitate was crystalline or amorphous. Formation of a highly soluble amorphous CaCO_3_ (ACC)^57^ would require that a high local supersaturation was reached in the gap between two dissolving calcite surfaces. This could have been possible in our system because of the increased solubility of the rough confining calcite surfaces and the limited diffusion in the confined solution. As proposed by Scherer^3^ (eq. 10 therein), we can estimate that the solubility of small, high-surface energy calcite crystals (with a radius of curvature = 10 nm) is ~1.5-fold higher than of large flat calcite crystals (assuming surface energy of calcite/water interface *γ* = 0.15 J/m^2^ ^14^). As such, we suggest that the metastable amorphous phase could have been formed in our system because of a lower thermodynamic barrier for its nucleation^58^. The expected recrystallization of ACC into calcite in our system was likely prevented due to the reduced ionic mobility in confinement. Such kinetic stabilization of ACC in confinement has been previously reported even in µm-thick gaps^36^.

The precipitate formed in the solution trapped between the surfaces, and was not strongly attached to the calcite surfaces. This was manifested in several ways: (1) we could visibly displace most of the newly-formed precipitate from the contact area when we approached the surfaces manually at very high loads ≫1 MPa (using the manual SFA micrometer control^40^; Fig. 4a–d); (2) by forcing the surfaces into contact at these very high loads (≫1 MPa), it was possible to reach the initial CP, which indicated no major change (<10 nm) of the calcite layer thickness in the contact region (Fig. S12); (3) we observed the changes in appearance of the FECO fringes: when the precipitate was present between the surfaces, the FECO fringes were very irregular; but when we squeezed the precipitate out the contact, the FECO fringes became regular again (Fig. 3e,f); (4) there was almost no change in calcite roughness measured at the end of the experiments, especially for the more uniform set 2 surfaces (Fig. S8). Unless we applied very high loads manually to squeeze the precipitate out of the contact, it remained between the surfaces until the end of the experiment (at applied loads <1 N/m ~ 0.5 MPa; Fig. S13).

As the position of the FECO fringes depends on the thickness and refractive index of each layer comprising the sample (in our case *Au-mica-calcite-solution-calcite-mica-Au*), it is possible to estimate the thickness of the precipitate in the contact region. We used an exemplary force-distance measurement after PF, in which the HP position (defined as the separation at a given applied load; Fig. 1b) was not changing significantly upon further increase in applied load. We assumed that this HP corresponded to the equilibrium thickness of the precipitate in the contact region at the given load (Fig. S15C,D; set 2, 0.1 M CaCl_2_). Assuming that the solution had the refractive index of water (${n}_{{H}_{2}O}$), the minimum separation between the surfaces after the PF at applied load of ~200 mN/m was ~650 nm (Fig. S15B). If there was a large difference between ${n}_{{H}_{2}O}$ and *n*_*precipitate*_, then this minimum separation could be largely overestimated. The dense precipitate likely had a higher *n* similar to a strongly hydrated ACC phase (*n*_*ACC*_ ~1.5)^59^. Using *n*_*ACC*_ ~1.5, the minimum separation between surfaces decreases to ~500 nm. Even if we used *n*_*calcite*_ ~1.65, the separation is >400 nm. This shows that the precipitate prevented the surfaces from coming into contact at moderate applied loads. A simple calculation, assuming a density of hydrated ACC (*ρ*~2.2 g/cm^3^)^60,61^, indicates that the precipitate could not have filled the entire volume between the surfaces (taking into account the maximum amount of Ca^2+^ from dissolving ALD calcite films, and the Ca^2+^ already present in the presaturated electrolyte solution), meaning that the precipitate must have been present as discontinuous domains or been of much lower density.

We never saw PFs initiating in the contact region established in the SFA, but rather propagating into the contact from outside the field of view. The region visible in our camera covers ~200 × 150 µm. This means that the observed PFs were initiated at distances >100 µm away from the location of the minimum surface separation. Due to the cylindrical geometry of our samples (*R* = 0.02 *m*), the surface separation (*D*) varies as a function of distance from the contact position (*x*) and can be approximated as $D=R-\sqrt{{R}^{2}-{x}^{2}}$^36^. The separation between two surfaces 100 µm away from the contact position is <0.3 µm. Earlier reports suggest that the influence of confinement on calcite crystallization can be present for surface separations <10 µm^36^. Then the ‘confined’ area (with radius of ~600 µm) in our SFA setup is 40 times larger than the nominal contact area. The largest separation between the surfaces is ~0.7 mm at the edges of the samples.

We have previously shown that dissolution of calcite in the SFA contact region is affected by the contact roughness^21^. In the current study, the initial dissolution of calcite before the PFs was also correlated with the surface roughness (as estimated from the initial CP at the beginning of the experiments^21^; Fig. S14C). However, we did not find any correlation between the time onset of PFs and neither the amount of dissolved calcite before PFs, nor the initial contact roughness (Fig. S14A,B). This may be related to the fact that the surface separation where the PFs were initiated was of the order of several µm. There, the nm-scale surface roughness of the calcite films should not additionally influence the transport of solutes along the gap.

As such, we interpret that PFs formed due to a local increase in the supersaturation with respect to the CaCO_3_ phase growing in the confined solution. The local supersaturation could have been reached due to the flux of ionic species from the dissolving confining surfaces. Because of the spatial confinement and the resulting limited diffusion out into the bulk solution, this supersaturation could have been maintained, and the nucleation was triggered. The location where the PFs originated likely depended on the local rates of dissolution of calcite surfaces and diffusion of the dissolved ionic species out of the gap: we suggest that the nucleation started outside of the contact regions as there the dissolution could have been faster (due to smaller confinement), but diffusion out into the bulk was still limited. PFs could spread into the observed contact regions as they followed the concentration gradients of the dissolved ionic species.

It is puzzling that the precipitation occurred in the solution confined between two surfaces and not by heterogenous nucleation onto the rough calcite surfaces. The rough surfaces contain plenty of favorable nucleation sites where the contact between the precipitating phase and the substrate would be large. However, if the interaction between the nucleating particle and the surface is repulsive, precipitation is energetically favored in the bulk solution^33^. Experimental and modelling studies have reported that highly coordinated water molecules on (104) calcite surfaces prevent direct adsorption of ionic species onto calcite surfaces and constitute an energetic barrier that may prevent heterogenous nucleation^62,63^. Moreover, surface-assisted nucleation on hydrophilic surfaces may be prevented if no oriented growth of mineral phase is promoted^64^. It is thus possible that heterogenous nucleation in our system is not favored as the calcite surfaces were strongly hydrated and repelled the precipitate and/or their nm-scale roughness prevented any oriented growth on the confining surfaces.

Interestingly, we observed nucleation of crystals in the contact region after the PF during repeated approaching and separation of the surfaces, when we moved the lower surface by means of the manual micrometer and repeatedly applied very high loads (>1 MPa; Fig. 4e–i; Supplementary Movies M16 and M18). Upon loading, most of the liquid-like precipitate was expelled from the contact region but it flowed back into the contact region on separation (Fig. 4a–d). After several in-out runs, we observed ~5 µm particles appearing between surfaces. These particles were flat (separation between the surfaces was <1 µm when surfaces were in contact; Fig. S18), loose (they were slightly changing position on the surface after each loading) and they first appeared when the surfaces were out of contact (Fig. 4e). Although we did not identify these particles undoubtedly (Fig. S17), they were very likely crystals of CaCO_3_ (the particles scattered light, making the FECO fringes discontinuous). It is therefore possible that at the very low supersaturation of our solutions, high loads had to be applied to squeeze the precipitate in a low volume-gap between the surfaces and, by repeatedly doing so, dehydrate the clustered ions^65^ and trigger crystallization. Pressure-induced phase transition due to dehydration has been previously observed for CaCO_3_^60^. Alternatively, by applying very high loads, we could have caused local pressure-induced dissolution of the highest asperities on the confining calcite surfaces, and therefore increase the local supersaturation to the level which allowed the growth of larger crystals. Although the growth of the larger crystallites at the expense of the liquid-like precipitate suspension can be explained by the Ostwald ripening process^66^, we never observed any spontaneous recrystallization of the liquid-like precipitate throughout the experiments (<25 h).

It is possible that the precipitate ripening during the SFA experiments was hampered due to confinement. If we assume that the equilibrium size of CaCO_3_ crystals growing between surfaces in the SFA is >1 µm, then stable crystals would not form unless they were able to displace the confining walls in order to reach that size. In the SFA setup, the top surface is fixed while the bottom surface is mounted on a force measuring spring with a spring constant *k* = 2000 N/m. Even if we did not apply any load to the bottom surface, the growing crystal would have to overcome a confining pressure of the order of MPa to displace the bottom surface by a distance (*x*) of several nm (for a 1 µm^2^ contact area), as estimated from *F* = −*kx*. Even at a very low supersaturation, the pressure exerted by growing calcite (calculated according to *Eq. 18* in Scherer^3^ assuming equilibrium solubility of calcite 0.0130 g/L and solute concentration of 0.0131 g/L) should be of a similar MPa order (~1 MPa). Therefore, the presence of confining walls in our setup should not hamper the growth of µm-sized crystals (we have previously observed formation of µm-sized crystals in the SFA setup with much more soluble ALD calcite films grown at lower temperatures^21^). We thus suggest that the precipitate ripening during the SFA experiments was prevented for two possible reasons: 1) dehydration of precipitate was hindered in the confinement due to reduced ion mobility; 2) the absolute amount of ionic species was insufficient to allow the growth of larger crystals.

### Influence of electrolytes on reactivity

**Figure 5:**
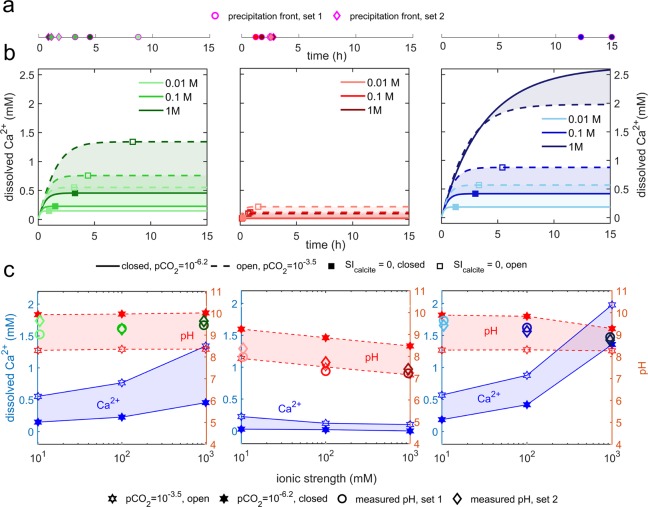
(**a**) Elapsed time before PFs measured for set 1 (○) and set 2 (◊) experiments; (**b**) Dissolution kinetics of calcite modelled in PhreeqC in NaCl, CaCl_2_ and MgCl_2_ solutions with IS = 0.01, 0.1, or 1 M for closed (low pCO_2_) and open (high pCO_2_) systems. Squares (□) represent SI_calcite_ = 0. The calculations were performed using the rate for calcite dissolution defined in the ‘*llnl.dat*’ database, assuming open (pCO_2_ = 10^−3.5^ atm) or closed systems (pCO_2_ = 10^−6.2^ atm). The rate equation is based on the model for calcite dissolution proposed by Plummer, *et al*.^78^. (**c**) Parameters of salt solutions used in the SFA. ○ and ◊ symbols show measured pH values of solutions used in set 1 and set 2 experiments. Highlighted areas show a range of possible pH and Ca^2+^concentrations for these solutions modelled in PhreeqC assuming low (closed system) and high (open system) partial pressure of CO_2_.

**Figure 6:**
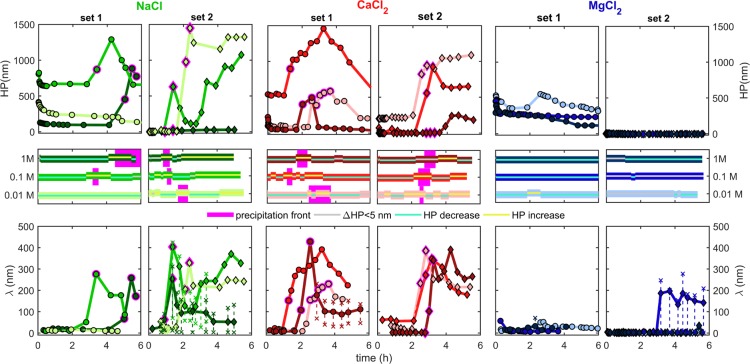
Evolution of hardwall position (HP) and exponential decay length of repulsive forces (λ) with time for SFA force measurements between two calcite surfaces in NaCl, CaCl_2_, and MgCl_2_ solutions with IS = 0.01, 0.1 and 1 M for set 1 and set 2 experiments (colors correspond to ionic strength as shown in the middle panel). The middle panel shows periods of HP increase or decrease, and duration of the precipitation fronts (PF) in the observed region on calcite surfaces. Data points measured during PFs are outlined with magenta. Red x symbols show a range of possible decay lengths, whenever exponential fits were poorly fit to the force-distance curves.

In Figs 5a and 6, we show the timing of the PFs as a function of electrolyte composition. The time elapsed before the onset of the PFs was found to be largest for the MgCl_2_/CaCO_3_ solutions (PFs observed after 12 and 15 h only for two experiments in set 1: 0.1 and 1 M), and shortest for experiments in CaCl_2_/CaCO_3_ (PFs were observed for all experiments within the initial 3 h). PFs for experiments in NaCl/CaCO_3_ occurred after 1 to 8 h. Precipitation in our experiments was a result of dissolution of the confining calcite surfaces and a subsequent increase in local supersaturation with respect to the CaCO_3_ phase growing in the confined solution. Since calcite dissolution and precipitation kinetics are known to be affected by the presence of background ions (due to: changes in ionic strength, ion-pair effects, ion solvation and a common-ion effect^24,25^), we expected to see the effect of salt composition and ionic strength on the timing of PFs.

Figure 5b illustrates the expected effect of ionic strength and solution composition on dissolution kinetics of calcite dissolving in NaCl, CaCl_2_ or MgCl_2_ solutions: using a simple PhreeqC model (Supplementary Information; section S10), we calculated the time and concentration of Ca^2+^ required to reach supersaturation with respect to calcite (SI_calcite_ = 0) in electrolyte solutions that initially contained no dissolved CaCO_3_. The modelling indicates that the lowest amount of dissolved calcite (expressed as Ca^2+^) and time are needed to saturate CaCl_2_ solutions (due to the common-ion effect that decreases solubility of CaCO_3_ in the presence of highly soluble CaCl_2_). Higher amount of dissolved calcite is needed to saturate NaCl and MgCl_2_ solutions (especially at MgCl_2_ with *IS* = 1 M due to the abundance of the MgHCO_3_^+^ ion pair that reduces the HCO_3_^−^ activity and shifts the calcite equilibrium).

The initial pH and composition of electrolyte solutions used in the SFA experiments is shown in Fig. 5c and Table S1. These solutions were saturated with respect to calcite (with SI_calcite_ ~0) and had pH values characteristic for saturation under low pCO_2_ conditions (Table S2). As the solutions were saturated, we should observe no dissolution of calcite surfaces in the SFA experiments and thus no precipitation. However, as discussed previously, the solubility of the ALD calcite surfaces was higher than the solubility of the calcite powder used for saturating the electrolyte solutions, which explains the initial dissolution of calcite films in the SFA experiments. We argued that during PFs the precipitate was formed in the confined solution. Therefore, we expected that the elapsed time before PFs would depend on solutions ionic composition and *IS* as illustrated in Fig. 5b: the onset of PFs should have been the fastest for CaCl_2_ solutions and the most delayed for the highest *IS* NaCl and MgCl_2_ solutions (we assume that the additional concentration of dissolved ions needed to reach supersaturation in our solutions would depend on the solution composition in a proportional manner as predicted by the PhreeqC modelling shown in Fig. 5b).

Although there was no clear correlation between the time onset of PFs and *IS* for any of the solutions (contrary to what was expected from calcite dissolution kinetics), we saw clear differences in time onsets of PFs for different salt solutions. PFs were the fastest in CaCl_2_/CaCO_3_ solutions (for all 6 experiments they occurred within the initial 3 h), which agrees with the reduced calcite solubility in the presence of CaCl_2_ due to the common ion effect. In turn, PFs were significantly postponed in MgCl_2_/CaCO_3_ solutions (they occurred for 2 out of six experiments after 12 and 17 h and never occurred for the set 2 one-day experiments), which shows importance of the ion-specific effects in our system. Inhibited nucleation of CaCO_3_ has been previously observed in the presence of Mg^2+^, with a higher threshold supersaturation needed to trigger mineral growth relative to Mg^2+^-free solutions^67^. Although there are several possible mechanisms by which Mg^2+^ modifies CaCO_3_ nucleation and affects the subsequent mineral growth (i.e. kinetic blocking of active growth sites due to Mg^2+^ adsorption^68^, reduced thermodynamic stability and increased solubility due to Mg^2+^ incorporation in the lattice^69,70^, or kinetic stabilization of metastable CaCO_3_ polymorphs^71,72^), there is a general agreement that Mg^2+^ hinders the nucleation of calcite and reduces calcite growth rates. This is in line with our findings, with greatly delayed PFs in the presence of Mg^2+^.

The lack of correlation between PFs onsets and ionic strength was likely related to the location on the sample where the PFs were initiated and the distance they propagated before we identified them in the camera. The fact that we observed PFs at various stages after they initiated, is supported by the differences in front velocities (~10 to ~500 nm/s) and the spreading manner (with full or partial coverage within the observed area), both likely resulting from the concentration gradients along the gap. As the separation between the surfaces continuously increases from the contact region towards the bulk solution, we expect both the differences in local dissolution rates of the calcite films and solute diffusion rate out of the gap to affect the time onset of PFs. We estimate that the uncertainty in the determination of actual PFs time onsets is <2 h: even though PFs were initiated outside the observed contact region, they still influenced the forces measured in a given contact. This is shown in Fig. 6 in which the magnitude and range of the repulsive forces (λ) had increased even before PF reached the contact region (we interpret this this increase in repulsion was due to nucleation and we associate maximum λ with the moment the PF reaches the observed contact, as discussed in the next section). Despite this uncertainty, we could still observe the distinct cation-specific effects on CaCO_3_ nucleation in the gap in CaCl_2_/CaCO_3_ and MgCl_2_/CaCO_3_ solutions.

### Long-range repulsive forces during and after precipitation

Both the hardwall positions (HP) and the magnitude and range of repulsive forces expressed as decay lengths (λ; see definitions in Fig. 1b) increased significantly during and after the *precipitation front* (PF) events. Figure 6 shows the changes in hardwall position (HP) and the corresponding changes in decay length λ with time for all 18 experiments. The decay lengths measured after PFs (45 nm < λ < 400 nm) were many times larger than before PFs (1 nm < λ < 65 nm). The location of HP for rough surfaces strongly depends on the applied load (Fig. S15C). We therefore determined the HP at the maximum load common to all measured force-distance curves in each experiment to clearly indicate major increases or decreases in HP as shown in Fig. 6. Additional parameters of the measured force curves (such as the maximum applied load and minimum separations at the maximum applied load) are shown in Fig. S13.

Figure 6 shows that in all experiments, the magnitude and range of repulsive forces (λ) were the smallest before PFs, and in all cases PFs were preceded by a period of calcite dissolution (indicated by decreases in HP ≪ 500 nm). We observed a clear correlation between the occurrence of PFs and decay lengths (λ): Whenever PFs reached the contact area, we measured a peak in repulsive forces (largest λ). If PFs occurred when surfaces were kept in contact under constant applied load, we found surfaces to move out of contact by tens of nm (set 1: 0.1 and 1 M MgCl_2_). This shows that the growing precipitate could act against loading and exert pressure on the confining walls. Although we could not determine whether the precipitate was amorphous or crystalline, this behavior shows that there was MPa-high pressure exerted by the growing CaCO_3_ phase^3^ associated with the precipitation fronts.

After the precipitation had ceased in the contact region, both the magnitude and onset of repulsive forces and HP gradually decreased but they were always at least one order of magnitude larger than at the beginning of each experiment (Fig. 6). In some experiments, HP after PF gradually reached the initial HP position measured at the start of experiments (set 1: 0.1 M NaCl, 0.1 M and 1 M CaCl_2_; set 2: 1 M NaCl; Figs 6, S13). We interpret such evolution of the repulsive forces to be caused by progressive depletion of the precipitate from the contact region upon repeated loading-unloading cycles (depletion could be also observed in the camera, e.g. Supplementary Movie M1). We additionally measured large hystereses between the force-distance curves on approach and retraction that were not present or were very small before the PFs. Areas of these hystereses closely followed the trend shown for the decay length of the repulsive force curves in each experiment: they were the largest during PFs and decreased with time after PFs. The presence of these hystereses indicate that there was an energy cost related to the displacement of the precipitate from the contact region. As areas of the hystereses became smaller with time, this additionally shows that the precipitate was progressively squeezed out from the contact region. Based on the above observations we interpret that the long-range, monotonically decaying and hysteretic repulsion measured after PFs was related to the hydrodynamic drag caused by the high viscosity of the precipitate^73^.

The precipitate trapped between the calcite surfaces was likely denser and more viscous than the bulk solution. Although the exact viscosity of the precipitate was unknown (and the effective viscosity of the confined solution was influenced by the inhomogeneous distribution of the clustered precipitate in the gap), high viscosity has been previously observed in colloidal suspensions of CaCO_3_ nanoparticles^74^. Due to viscous forces, the precipitate would oppose the movement of the surfaces (similarly to what has been previously observed in SFA force measurements with non-adsorbing polymer melts^73^), giving rise to repulsive force on approach and hystereses between loading-unloading force curves. Assuming no-slip conditions, the hydrodynamic force *F*_*h*_is proportional to the movement velocity *v* and fluid viscosity *η*, and can be estimated as ${F}_{h}=\frac{6\pi \eta {R}^{2}v}{D}$ (for the crossed cylinder geometry of the SFA), where D is separation between the surfaces, R is cylinder radius, and D ≪ R^75^. We did not observe any correlation between the magnitude and onset of the measured repulsive force and the approach velocity in our experiments, something that should be present if hydrodynamic effects were at play. However, the range of the velocities that we used after PFs (~1 to 5 nm/s) could be insufficient to observe significant differences in the hydrodynamic contribution, especially with an inhomogeneous distribution of the viscous phase in the gap.

Long-range repulsion could additionally arise due to entropic, steric effects that are related to the confinement of the denser phase between the surfaces^54^. If the loading was too fast for the precipitate to be displaced from the gap, it could have become partially jammed between surfaces. Such trapped precipitate would oppose the surface movement either because energy was needed for its progressive dehydration or there was little available volume for its spatial rearrangement. We observed that after PFs and after several loading-unloading cycles some sort of CP was developing at large separations (hundreds of nm away from the initial CP, Fig. S15D), where separation did not decrease despite further loading (e.g. set 2, 0.1 M CaCl_2_ experiment, Fig. 6). This reflected a high energy cost both to displace more precipitate from the gap and to further squeeze it in the gap (the range of applied loads that we used during the force measurements is plotted in Fig. S13).

We have previously performed a series of similar SFA experiments, in which we used CaCO_3_-presaturated solutions without added electrolytes^21^. We only observed major increases in the magnitude and onset of repulsive forces in a few experiments, in which the roughness of the surfaces was the smallest, and we have attributed this increased repulsion to the recrystallization of calcite surfaces. The findings of the current study, where we have performed a more thorough analysis of the roughness change after the experiments and we could observe reproducible PFs in almost all experiments, suggest that the increase in magnitude and range of the repulsive forces measured in the previous study was also likely related to CaCO_3_ nucleation in the confined solution. The electrolyte solutions used in the current study speeded up the occurrence of PFs (either due faster dissolution of films in high-*IS* NaCl solutions or decreased calcite solubility in CaCl_2_ solutions). Thus, it was easier to trigger the PFs (even for the rougher contacts in the set 1 experiments), which required that the confined solution became locally supersaturated with respect to the nucleating CaCO_3_ phase.

## Conclusions

We showed that properties of the solution confined between two reactive calcite surfaces can affect interfacial forces even at µm-ranged surface separations. We observed nucleation of submicron-sized precipitate that formed in the confined solution, in which local supersaturation with respect to the nucleating phase was attained due to the initial dissolution of the confining surfaces. The viscous precipitate, which was most likely an amorphous hydrated CaCO_3_ phase, gave rise to long-range and high-magnitude repulsion acting between two calcite surfaces. These observations may have crucial consequences for the evolution of microstructure of both fluid-saturated rocks and mineral-based materials: (1) We measured the long-range repulsive forces at ionic strengths varying from 0.01 to 1 M. This shows that the strengthening of solid-solid contacts at high ionic strengths, as expected from the DLVO theory due to the dominance of attractive force contributions, can be counteracted by nucleation occurring in the solution confined between two solid surfaces; (2) The onset of nucleation was influenced by ion specific effects to a higher extent than by the solution ionic strength, with Mg^2+^ significantly delaying the nucleation. This demonstrates the importance of ion-specific effects for confined crystallization; (3) Although we did not measure diffusion rates of ionic species between the two calcite surfaces in our experiments, we suggest that the transport of reactants between mineral surfaces can be significantly slowed down in the presence of the dense precipitate that we observed, even at µm-range separations. Although it is generally expected that the diffusion coefficient of ionic species in confined solution should not be affected for separations larger than a few nm, we showed a possible mechanism that can delay diffusion in relatively thick gaps. (4) Our measurements indicate that at the timescale of our experiments cementation of grain interfaces is not likely to proceed at low supersaturation conditions, as there exists an energy barrier for dehydration of the precipitate nucleating in confinement, even when the gaps between surfaces are µm-thick. We also suggest that cementation may be hampered if the interaction between the cementing phase and the confining walls is repulsive, as it prevents the heterogenous nucleation on the confining walls; (5) We showed that the occurrence of precipitation fronts in our system was correlated with the repulsive forces of the highest magnitudes. Therefore, the significant mechanical repulsion related to the pressure exerted on the confining walls can be present even when the nucleating phases are composed of submicron-sized particles. Future work should involve more precise, *in situ* characterization of the nucleating phase.

## Methods

### Preparation and characterization of calcite films

Thin (~200 nm), polycrystalline calcite films were grown at 300 °C by Atomic Layer Deposition (ALD) as described in Nilsen, *et al*.^46^ using a F-120 Sat reactor from ASM Microchemistry. The detailed preparation of the calcite films deposited on mica substrates for the SFA has been explained in Dziadkowiec, *et al*.^21^. Because of substantial variation in roughness of ALD calcite films, we prepared 3 sets of surfaces, each in a separate ALD run. Sets 1 and 2 were used for the SFA measurements and set 3 was used for the AFM measurements in salt solutions. Detailed deposition and film parameters are provided in Supplementary Information (section S5). After the deposition, calcite films were kept in a vacuum-sealed desiccator.

X-ray diffraction (XRD) was used to identify the ALD-grown CaCO_3_ phase on Au-coated glass slides (XRD peaks of mica substrate overlapped with the most intense calcite peak). We used Bruker AXS D8 Discover powder diffractometer in Bragg-Brentano configuration, equipped with a Lynxeye detector, using Cu Kα1 radiation and a Ge(111) monochromator.

Film morphology was observed with Scanning Electron Microscopy (SEM), using Hitachi SU5000 FE-SEM in secondary electrons (SE) mode (15 kV). The samples were coated with ~3 nm of Au.

Film topography was analyzed in air with AFM (JPK NanoWizard®4 Bioscience), in QI-mode before and after the SFA experiments. A ContAl-G cantilever (NanoSensors, k = 0.2 N/m and l = 450 µm) was used to scan the surfaces (scan sizes of 2 × 2 and 15 × 15 µm^2^. Both for SEM and AFM, the samples used in the SFA were quickly dried with N_2_ after the experiments. The samples observed after the SFA experiments appeared cracked, but the cracking was caused by fast sample drying in a laminar flow cabinet (we would also observe such large cracks in the SFA camera if they appeared during the experiments).

### SFA measurements and data analysis

Nm-range forces between two rough and polycrystalline calcite surfaces were measured with the SFA (SFA2000; SurForce LLC, USA^40^) as a function of a distance between the surfaces. Our SFA is coupled with MBI (Princeton Instruments IsoPlane SCT320 spectrometer and a PIXIS2048B camera with a lateral resolution of 0.62 µm/pixel), and a Thorlabs DCC1645C camera (0.15 μm/pixel resolution) aiding surface topography observation. The spectrometer was calibrated using an Hg lamp within a 520–630 nm spectral range and spectrometer gratings of three different resolutions (600, 1200, 1800 g/mm) were used, depending on the mica substrate thickness. MBI provides information about surface separation and topographic information *in situ* through the FECO fringes, which are sensitive to thickness and refractive index of the sample^43^. Calcite surfaces on mica substrate were glued to cylindrical glass disks with the radius of curvature R = 2 cm, which yielded nominal contact areas of 100–150 µm in diameter. The bottom surface was mounted on a force measuring spring, with a spring constant k = 2000 N/m. The principles of the SFA and MBI techniques have been described in^40,42,43,76^. For each experiment we used two fresh pieces of the ALD-deposited calcite films. We first established a suitable contact area, without visible, larger surface asperities, estimated the thickness of calcite surfaces, and then measured forces in the same contact throughout the 2-days (set 1) or 1-day (set 2) experiments. We analysed the SFA data using the open source Reflcalc software^77^, which can simulate the FECO fringe patters by calculating the light transmission through our multi-layered samples. Identification of FECO wavelength positions and data processing has been handled in the MATLAB software. The details of Reflcalc modelling, data analysis, and typical experimental steps have been outlined in Dziadkowiec, *et al*.^21^ and the Supplementary Information therein. We expect a relatively small error in determination of absolute separation between the surfaces for experiments in which we observed flattening of FECO fringes in contact (<20 nm, set 2 before PFs), and larger errors for rougher surfaces where the contact position was not reached in the range of applied loads that we used (even ~100 nm)^21^. The relative error between the consecutive data points in force curves, due to misestimation of absolute separation, should be less than several nm^21^.

### Atomic force microscopy (AFM) measurements

Roughness evolution with time of single, unconfined calcite films in salt solutions was analyzed with the Atomic Force Microscope (AFM; MFP3D, Asylum Research, Oxford Instrument). A soft, uncoated quartz-like AFM tip with k = 0.01 N/m (qp-SCONT; NANOSENSORS™ uniqprobes) was used to image the surfaces in a contact mode (scan size 3 × 3 μm^2^, resolution of 512 pixels). The experiments were carried out in stationary salt solutions, in a homemade, non-sealed fluid cell with a volume of ~3 ml. We thus observed some evaporation during the experiments, leading to an increase in salt concentration throughout the experiment. In each experiment we continuously scanned the same position on the film surface, however due to instrumental drift we usually observed a μm-range shift from the initial scan position. A new piece of calcite film deposited on mica (ALD set 3) was used for each experiment.

### Solutions

We used NaCl, CaCl_2_ and MgCl_2_ salt solutions with ionic strength of 10, 100 and 1000 mM. All solutions were presaturated with calcite by adding ~1 g/L of synthetic calcite powder (Merck KGaA; baked at 300 °C for 2 hours before use to reduce possible organic contamination). The salt/CaCO_3_ solutions were sealed and stirred for more than one week prior to use. Prior to the SFA and AFM experiments, all solutions were filtered with 0.2 µm polyether-sulfone filters and injected into the SFA (~150 ml) or AFM (~3 ml) directly after filtration. In the SFA the solutions were injected when keeping the two calcite surfaces in contact to limit dissolution upon equilibration with the solution. Every time when a new solution was injected into the SFA, the SFA chamber was drained with an excess ~150 ml of the same solution to limit possible contamination. The saturation indices (SI) with respect to calcite and Ca^2+^ concentration were calculated in the PhreeqC software, using the ‘*llnl.dat*’ database, based on the measured pH and assuming pCO_2_ both for closed (10^−6.2^ atm) and open systems (10^−3.5^ atm).

## Supplementary information

Supplementary Information

Supplementary movie M1

Supplementary movie M2

Supplementary movie M3

Supplementary movie M4

Supplementary movie M5

Supplementary movie M6

Supplementary movie M7

Supplementary movie M8

Supplementary movie M9

Supplementary movie M10

Supplementary movie M11

Supplementary movie M12

Supplementary movie M13

Supplementary movie M14

Supplementary movie M15

Supplementary movie M16

Supplementary movie M17

Supplementary movie M18

## Data Availability

The datasets generated during the current study are available from the corresponding author on reasonable request.
