# Supplementary material for: Nucleation in confinement generates long-range repulsion between rough calcite surfaces
Source: Sci Rep. 2019 Jun 20;9:8948. doi: 10.1038/s41598-019-45163-6 (PMC6586869; doi:10.1038/s41598-019-45163-6)
Supplement: Supplementary file 1 — Supplementary Information [file 41598_2019_45163_MOESM1_ESM.pdf]

Supplementary Information for

Nucleation in confinement generates long-range repulsive forces between rough calcite surfaces.

Joanna Dziadkowiec*[,1], Bahareh Zareeipolgardani[2], Dag Kristian Dysthe[1], Anja Røyne[1]

[1]Physics of Geological Processes (PGP), The NJORD Centre, Department of Physics, University of Oslo, Oslo 0371, Norway

[2]Institut Lumière Matière, Université de Lyon, Université Claude Bernard Lyon 1, CNRS UMR 5586, Campus de la Doua, F-69622 Villeurbanne cedex, France

* joanna.dziadkowiec@fys.uio.no

S1. Supplementary Movies

The field of view is 192 µm x 154 µm for the movies M1-M15. Magnification of the M16 movie is indicated with a scale bar. Time is displayed with respect to injection times of electrolyte solutions into the SFA chamber.

FR – the movie was recorded during force measurements (continued loading-unloading cycles)

TO – the movie was recorded when the surfaces were kept in contact under constant load

PF onset – elapsed time after the solution injection when the precipitation front (PF) entered the observed region

Movies showing precipitation fronts: Set 1 experiments

Movie 1: 0.01 M NaCl experiment ('M1_NaCl001M_set1.avi')

PF onset: 9 h; TO.

Note a depletion of the precipitate in the observed region after 15h, and a second PF event after 17 h.

Movie 2: 0.1 M NaCl experiment ('M2_NaCl01M_set1.avi')

PF onset: 3 h 30 min; FR.

Movie 3: 1 M NaCl experiment ('M3_NaCl1M_set1.avi')

PF onset: 4 h 20 min; FR.

Movie 4: 0.01 M $CaCl_2$ experiment ('M4_CaCl001M_set1.avi')

PF onset: 2 h 18 min; FR.

The PF event was captured in the observed region only at its initial stage.

Movie 5: 0.1 M $CaCl_2$ experiment ('M5_cacl01M_set1.avi')

PF onset: 2 h 20 min; FR.

Movie 6: 1 M $CaCl_2$ experiment ('M6_cacl1M_set1.avi')

PF onset: 1 h 40 min; FR.

Movie 7: 0.1 M $MgCl_2$ experiment ('M7_mgcl01M_set1.avi')

PF onset: 12 h; TO.

Movie 8: 1 M $MgCl_2$ experiment ('M8_mgcl1M_set1.avi')

PF onset: 14 h 30 min; TO.

Movies showing precipitation fronts: Set 2 experiments

Movie 9: 0.01 M NaCl experiment (‘M9_NaCl001_set2.avi’)

PF onset: 1 h 40 min; FR.

Movie 10: 0.1 M NaCl experiment (‘M10_NaCl01_set2.avi’)

PF onset: 1 h 20 min; FR.

Movie 11: 1 M NaCl experiment (‘M11_NaCl1_set2.avi’)

PF onset: 50 min; FR.

Precipitate forms discontinuous domains and is mobile on repeated loading-unloading cycles.

Movie 12: 0.01 M $CaCl_2$ experiment (‘M12_CaCl001_set2.avi’)

PF onset: 48 min; FR.

Movie 13: 0.1 M $CaCl_2$ experiment (‘M13_CaCl01_set2.avi)

PF onset: 2h 10 min; FR.

Movie 14: 1 M $CaCl_2$ experiment (‘M14_CaC1_set2.avi)

PF onset: 2h 40 min; FR.

Movie 17: 0.1 M NaCl experiment – control, saturated in an open system (pCO2 = $10^{-3.5}$ atm) (‘M17_nacl01M_open.avi)

PF onset: 4h 13 min; FR.

Movie showing a full loading-unloading cycle after the PF

Movie 15: 0.1 M NaCl experiment, set 1 (‘M15_run_afterPF.avi’)

SFA force measurement 26 h after the solution injection and 22 h 30 min after the PF.

Movie showing smashing of the precipitate

Movie 16: precipitate smashing (‘M16_precipitate_smashing.avi’)

Movie 18: precipitate smashing (‘M18_precipitate_smashing.avi’)

Surfaces were approached at very high loads using the manual SFA micrometer control. After several loading-unloading cycles, µm-sized spherical particles started to appear in the contact region. It was very likely that these particles represented $CaCO_3$ crystals. Crystals are not attached to the surfaces and move when the calcite surfaces are separated from each other.

S2. Solutions used in the SFA experiments

Set	Salt	Ionic strength (mM)	Duration (days)	Measured pH		Calculated pH (PhreeqC)	
				initial	final	closed system	open system
**1**	NaCl/ $CaCO_3$	1000	2	9.45	9.13	10.01	8.36
**2**	NaCl/ $CaCO_3$	1000	1	9.64	9.58		
**1**	NaCl/ $CaCO_3$	100	2	9.30	9.03	9.96	8.35
**2**	NaCl/ $CaCO_3$	100	1	9.27	9.22		
**2**	NaCl/ $CaCO_3$ (open control)	100	1	8.39	8.35		
**1**	NaCl/ $CaCO_3$	10	2	9.03	8.70	9.94	8.30
**2**	NaCl/ $CaCO_3$	10	1	9.65	9.42		
**1**	$CaCl_2$/$CaCO_3$	1000	2	7.22	7.63	8.47	7.15
**2**	$CaCl_2$/$CaCO_3$	1000	1	7.41	7.17		
**1**	$CaCl_2$/$CaCO_3$	100	2	7.32	8.47	8.85	7.51
**2**	$CaCl_2$/$CaCO_3$	100	1	7.72	7.15		
**1**	$CaCl_2$/$CaCO_3$	10	2	7.97	8.70	9.24	7.90
**2**	$CaCl_2$/$CaCO_3$	10	1	8.35	8.03		
**1**	$MgCl_2$/ $CaCO_3$	1000	2	8.89	8.6	9.27	8.26
**2**	$MgCl_2$/ $CaCO_3$	1000	1	8.75	8.68		
**1**	$MgCl_2$/ $CaCO_3$	100	2	9.32	9.24	9.84	8.31
**2**	$MgCl_2$/ $CaCO_3$	100	1	9.15	9.05		
**1**	$MgCl_2$/ $CaCO_3$	10	2	9.64	9.1	9.89	8.29
**2**	$MgCl_2$/ $CaCO_3$	10	1	9.42	9.27		

Table S1. *Parameters of salt solutions saturated with respect to $CaCO_3$ used in the Surface Forces Apparatus (SFA) experiments. Parameters of solutions used in set 1 and set 2 experiments have been indicated. The pH of the solutions was measured just before the injection into the SFA chamber (initial pH) and at the end of experiments (pH final). The change of solutions' pH is more significant for the 2-day experiments. The table shows also expected pH values of the solutions calculated in the PhreeqC software (calculated pH), assuming that solutions undergo saturation with respect to calcite in an open system with respect to CO_2 (pCO2 = $10^{-3.5}$ atm) and in a closed system (pCO2 = $10^{-6.2}$ atm). The measured pH was always in between the theoretical pH values calculated for the open and closed systems, as illustrated in Figure 5c in the main manuscript.*

salt	IS (mM)	set	PhreeqC modelling - initial solution				PhreeqC modelling - final solution				ΔCa initial and final solution (mM)	$SI_{calcite}$ (-) of final solution (assuming no calcite could dissolve)
			initial measured pH	total dissolved Ca (mM)	total dissolved C(4) (mM)	pCO_2 (atm)	final measured pH	total dissolved Ca (mM)	total dissolved C(4) (mM)	pCO_2 (atm)		
NaCl	10	1	9.03	0.25	0.46	10^-4.63	8.7	0.36	0.69	10^-4.1	0.11	-0.42
		2	9.65	0.16	0.22	10^-5.65	9.42	0.18	0.29	10^-5.25	0.02	-0.15
	100	1	9.30	0.30	0.48	10^-5.02	9.03	0.37	0.67	10^-4.57	0.08	-0.26
		2	9.27	0.30	0.50	10^-4.97	9.22	0.32	0.53	10^-4.88	0.01	-0.05
	1000	1	9.45	0.53	0.76	10^-5.28	9.13	0.65	1.07	10^-4.73	0.12	-0.22
		2	9.64	0.49	0.63	10^-5.63	9.58	0.50	0.67	10^-5.51	0.01	-0.02
$CaCl_2$	10	1	7.97	3.51	0.36	10^-3.66	8.7	3.38	0.08	10^-5.1	-0.14	0.94
		2	8.35	3.41	0.16	10^-4.41	8.03	3.49	0.32	10^-3.77	0.08	-0.56
	100	1	7.32	33.52	0.40	10^-3.1	8.47	33.35	0.03	10^-5.38	-0.17	1.23
		2	7.72	33.41	0.15	10^-3.92	7.15	33.60	0.59	10^-2.78	0.19	-1.05
	1000	1	7.22	333.40	0.17	10^-3.65	7.63	333.40	0.07	10^-4.46	0.00	0.56
		2	7.41	333.40	0.11	10^-4.05	7.17	333.40	0.19	10^-3.55	0.00	-0.42
$MgCl_2$	10	1	9.64	0.19	0.24	10^-5.73	9.1	0.26	0.43	10^-4.78	0.07	-0.33
		2	9.42	0.21	0.30	10^-5.32	9.27	0.23	0.36	10^-5.06	0.02	-0.09
	100	1	9.32	0.45	0.57	10^-5.24	9.24	0.46	0.61	10^-5.1	0.01	-0.02
		2	9.15	0.48	0.66	10^-4.93	9.05	0.51	0.72	10^-4.75	0.02	-0.05
	1000	1	8.89	1.40	1.81	10^-4.63	8.6	1.59	2.33	10^-4.08	0.19	-0.13
		2	8.75	1.48	2.03	10^-4.36	8.68	1.53	2.16	10^-4.23	0.05	-0.03

Table S2. *Parameters of the solutions used in the SFA measurements calculated with PhreeqC software based on the pH values measured at the beginning and end of each experiment. All solutions were saturated with respect to calcite (saturation index is $SI_{calcite}$ ~ 0). IS - ionic strength. The final pCO_2 were calculated based on the assumption that the changes in measured pH were driven only by the additional dissolution of CO_2 in the solutions. The salt solutions used in the SFA experiments were equilibrated with calcite powder under $pCO_2 < 10^{-3.5}$ atm (closed volumetric flasks) in order to limit any possible contamination. Saturation under pCO_2 < 10-3.5 atm, means that some minor amount of CO_2 from the atmosphere (pCO_2 ~ 10-3.5 atm) can additionally dissolve into the solutions inside the SFA chamber during the experiments. However, as the chamber is sealed, this extra dissolution of CO_2 in the solutions is not major (as demonstrated by the final pH measured at the end of experiments, which pH values are still above the pH values calculated for an open system; see **Table S1**). We estimate here that the additional dissolution of CO_2 in the solutions during the experiments can drive a slight dissolution of calcite upon equilibration with the solution. This results in the < 0.2 mM-increase in the concentration of the dissolved Ca species. The main driving force for ALD calcite dissolution is, however, surface roughness and the presence of high-energy faces on some of the crystals comprising the deposited ALD calcite surfaces (see **Figure S1** and section **S3**).*

S3. Gibbs free energy change calculations

To quantify the magnitude of driving forces for dissolution and recrystallization of rough ALD calcite surfaces in our system we estimated the following changes in Gibbs free energy (ΔG) relative to flat calcite:

1) ΔG due to the possible presence of calcite crystals with high energy, highly soluble faces. We previously observed that crystals with high-energy faces comprise ALD films grown at a lower temperature (grown at 250°C in comparison with 300°C used in this study) and that these low-temperature films immediately dissolve in contact with saturated $CaCO_3$-solutions[1]. Some small proportion of the crystals with high-energy faces may also be present in ALD calcite films grown at higher temperatures[1,2]. We therefore calculated ΔG of high-energy calcite crystals in ALD films by assuming that the solubility product of these crystals at 25°C ($K_{sp,ALD-high}$) is 3 to 5 times larger than the solubility product of smooth calcite[3] $K_{sp,calcite} = 3.3 \cdot 10^{-9}$, using the formula $\Delta G = RTln(K_{sp,ALD-high}/K_{sp,calcite})$;

2) ΔG due to high roughness of ALD calcite surfaces. ΔG was calculated from Laplace pressure of small crystals with a high surface curvature, using the expression $\Delta G = \frac{2\gamma\bar{V}}{r}$, where γ= 0.15 J/m^2 is the interfacial energy of a calcite/water interface [4], $\bar{V}$ = 36.4 cm^3/mol is molar volume of calcite, and r is radius of crystal curvature. Based on the SEM images and AFM topography maps of the ALD calcite surfaces we chose the r values between 10 and 50 nm (corresponding to two dashed lines in Figure S1);

3) ΔG due to changes in saturation with respect to calcite upon dissolution of CO_2 into the electrolyte solutions during experiments. ΔG was calculated from $\Delta G = \Omega RT$, where Ω is saturation index with respect to flat calcite computed with the PhreeqC code (see section S8). To estimate changes in Ω due to CO_2 diffusion into the solutions, we performed a control test in which we placed the 0.1 M NaCl/$CaCO_3$ solution (saturated with calcite under closed conditions, initial pCO_2 = $10^{-5.76}$ atm, pH = 9.72) inside the sealed SFA chamber without any calcite surfaces and measured the evolution of pH within 20 hours. In this case, the drop in pH corresponds solely to the diffusion of CO_2 into the solution as there are no calcite surfaces to equilibrate the solution anymore. As we measured most of the precipitation fronts within first 6 hours, we used the pH value measured in this control after 7 h (9.42) to calculate the undersaturation of the solution due to extra CO_2 dissolution. We also used the final pH value measured after 20 h (9.31). The undersaturation was Ω = -0.13 (after 7 h) and Ω = -0.19 (after 20 h) as calculated in PhreeqC. The corresponding ΔG due to changes in saturation was -0.3 kJ/mol (after 7 h) and -0.5 kJ/mol (after 20 h) as shown in **Figure S1** with two cyan lines). Please note that these changes in pCO_2 are overestimated as each time we took a 5 ml sample of the solution we had to open the SFA chamber, and the SFA chamber was not completely filled with the solution anymore; during the experiments, we never open the SFA chamber and it is completely filled with the solution.

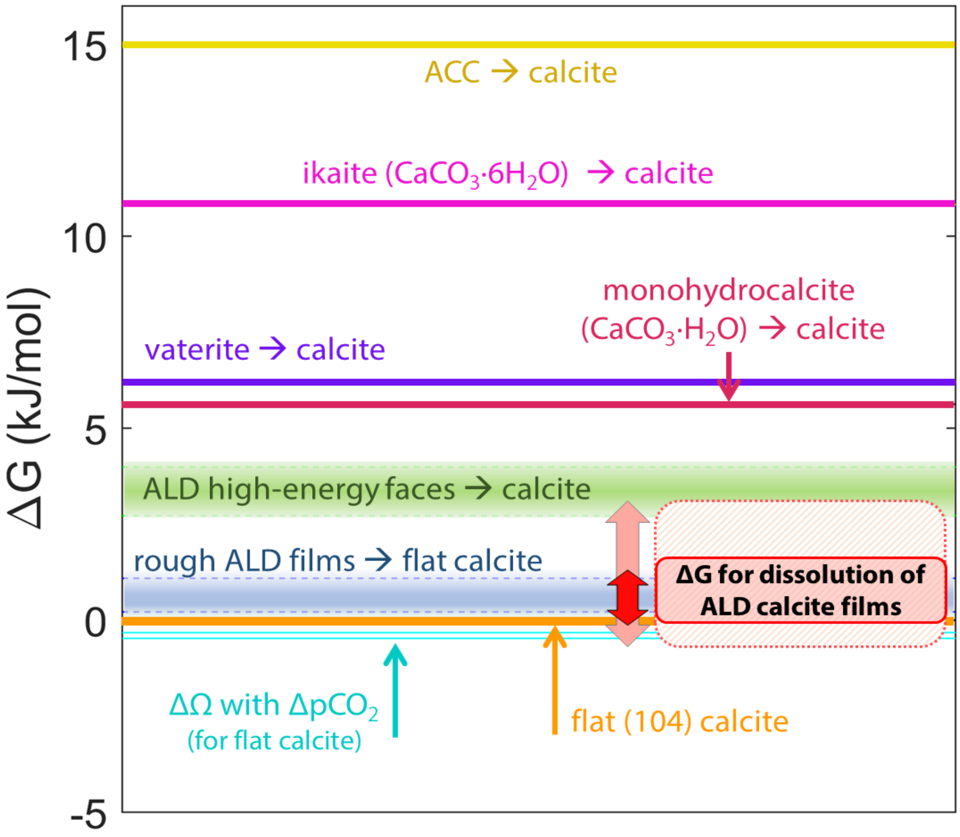

Figure S1. *Schematic representation of changes in Gibbs free energy (ΔG) with respect to the most stable flat (104) calcite face, estimating the thermodynamic driving forces for dissolution and recrystallization of rough ALD calcite in our system. We compare ΔG of various $CaCO_3$ phases (amorphous calcium carbonate - ACC[5], ikaite[6], vaterite[7], and monohydrocalcite[8]) and ΔG of rough ALD calcite, as well as ΔG due to the changes in solution saturation with respect to calcite (Ω) with the changing pCO_2 during the SFA experiments. The estimated ΔG values show that the main driving force for the dissolution and recrystallization of ALD calcite in our system is a high curvature of calcite crystals comprising the rough ALD calcite films. ΔG of the rough ALD calcite films can further increase due to the presence of a minor amount of calcite crystals having high energy, highly soluble faces (different than the most stable (104) calcite faces) in the ALD films[9] (ALD high-energy faces region). The changes in Gibbs free energy due to the ΔΩ of the solutions upon the limited diffusion of pCO_2 into the SFA chamber are of a smaller magnitude (two cyan lines correspond to ΔΩ after 20h – lower ΔG, and after 7h – higher ΔG). This means that the additional dissolution of CO_2 in the solutions can further decrease the lower bound of the free energy region available for dissolution and recrystallization of the rough ALD calcite surfaces (marked with a red arrow), but it is not the main driving force of this process. High ΔG of amorphous and hydrated $CaCO_3$ with respect to calcite indicates that if amorphous or hydrated $CaCO_3$ phase nucleated between calcite surfaces during precipitation fronts in our SFA experiments, it is thermodynamically unstable, and its persistence can be explained by kinetic stabilization in confinement.*

S4. Control experiment with solution saturated with calcite at $pCO_2 = 10^{-3.5}$ atm (open system)

A control experiment was performed with 100 mM NaCl solution saturated with calcite until fully equilibrated with atmospheric pCO_2 ($pCO_2 \approx 10^{-3.5}$ atm). The goal of this control was to demonstrate that the possible diffusion of extra CO_2 into the sealed SFA chamber when using solutions saturated with calcite in a closed system ($pCO_2 < 10^{-3.5}$ atm) is not the main driving force for dissolution of calcite surfaces and for the subsequent occurrence of the precipitation fronts in our experiments. The precipitation front also occurred in this control experiment, and the timing of the precipitation front (after 4 h since the solution injection) was similar to the timing of the precipitation fronts measured for the 100 mM NaCl solutions saturated in a closed system (see **Figure 5a** in the manuscript). This indicated that the estimations of thermodynamic driving forces for calcite dissolution shown in Figure S1 are correct and that the extra diffusion of CO_2 into the solutions inside the SFA chamber is not the main driving force for the occurrence of the precipitation fronts in our experimental setup.

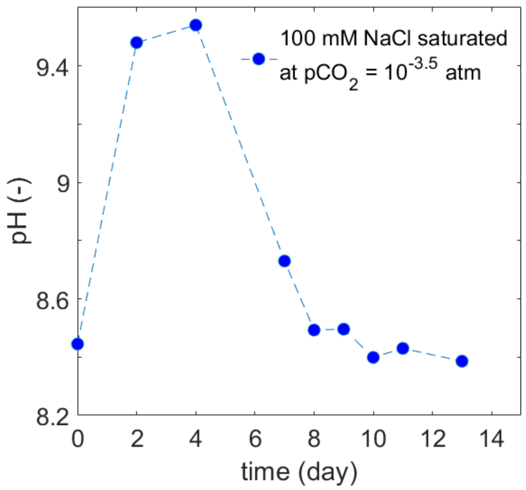

Figure S2. *Evolution of pH during saturation of 100 mM NaCl solution with calcite powder in open conditions ($pCO_2 \approx 10^{-3.5}$ atm). The solution was kept in an open flask and was constantly and vigorously stirred (1000 rpm). Before each pH measurement, ~10 ml of the solution was filtered (0.02 µm) to remove suspended calcite particles. The solution was used in the control SFA experiment after 13 days of saturation (pH 8.386). pH at the end of SFA control experiment was 8.35 (after 6 h) and 8.33 (after 20 h).*

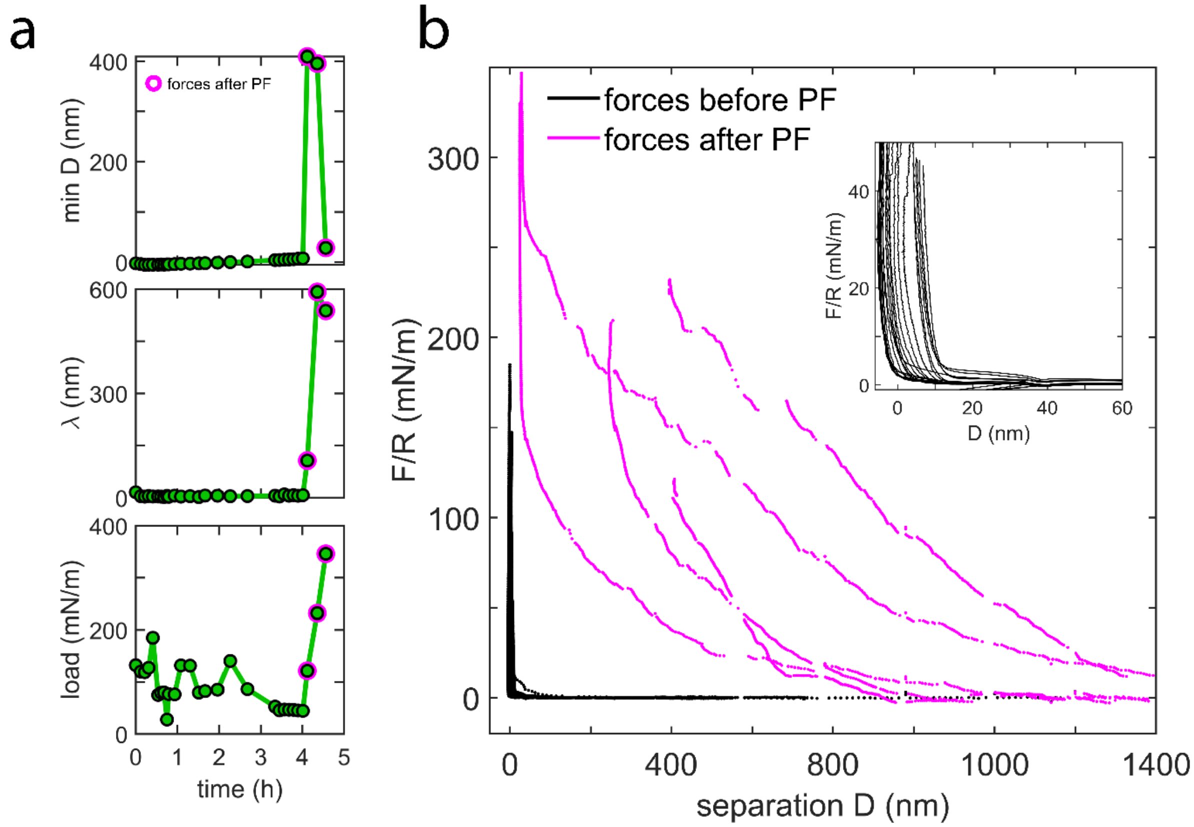

Figure S3. *Results of the SFA control experiment, in which forces between two calcite (set 2) surfaces were measured in 100 mM NaCl solution saturated with calcite under open conditions (pH = 8.39; see Figure S2). In this experiment, the extra diffusion of CO_2 into solutions is almost completely eliminated as the solution was in equilibrium with atmospheric pCO_2. Similar to experiments performed with solutions saturated under closed conditions, we observed an occurrence of a precipitation front (PF). The PF timing (4 h after the solution injection) was comparable with other experiments in 100 mM NaCl solutions (Figure 5 in the manuscript). a) Minimum separation between surfaces, exponential decay length (λ) of the force curves measured on approach and maximum applied load as a function of time. Each data point corresponds to one force measurement. Both minimum separation between surfaces and λ (proportional to the range and magnitude of the measured repulsive forces) substantially increased after 4 hours, which was correlated with the occurrence of PF (Supplementary Movie M17_nacl01M_open.avi). b) All force-distance (D) curves measured during the SFA control experiment. The black lines show forces measured before PF (zoomed in in the inset), and the magenta lines show forces measured during or after the PF. The presence of viscous precipitate in the solution between calcite surfaces gave rise to a substantial increase in the range and magnitude of the repulsive forces between two calcite surfaces.*

S5. Atomic Layer Deposition (ALD) Parameters

Table S3. *ALD deposition parameters for the set 1, set 2 and set 3 calcite surfaces grown on mica substrates using the F-120 Sat reactor from ASM Microchemistry by the procedure adapted from Nilsen, et al. [2]. The Ca^{2+} organic precursor was $Ca(thd)_2$ (Volatec; 97%; Hthds = 2,2,6,6-tetramethylheptan-3,5-dione).*

T (˚C)	aimed thickness (nm)	sublimation T (˚C)	deposition cycles						number of cycles
			$Ca(thd)_2$ pulse (s)	N_2 purge (s)	O_3 pulse (s)	N_2 purge (s)	CO_2 pulse (s)	N_2 purge (s)	
300	100	195	3	2	3	2	3	2	2000

S6. X-ray Diffraction (XRD)

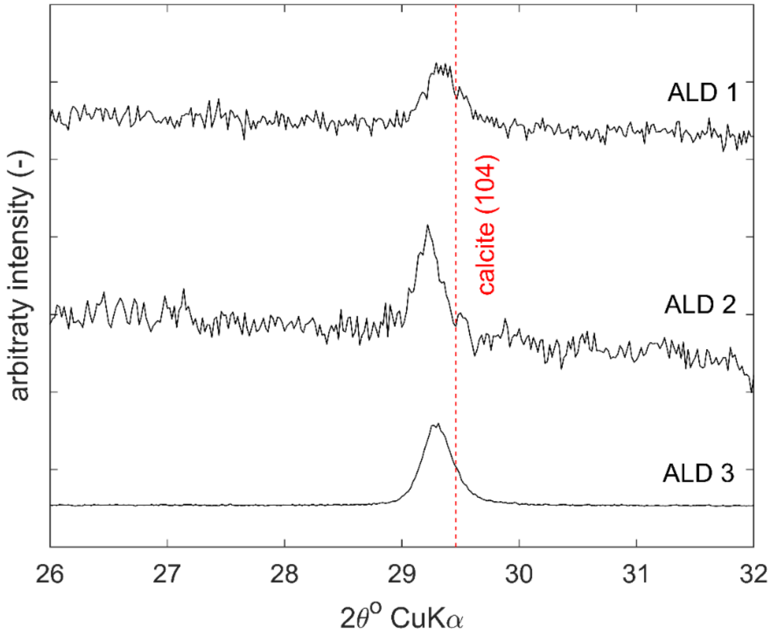

Figure S4. *XRD of ALD-deposited calcite films on Au substrate (ALD 1 - set 1, ALD 2 – set 2) and on Si wafer substrate (ALD 3 used in AFM; Figures S6, S7). Only the most intense calcite (104) peak can be identified for the films due to their small thickness (~100 nm). The position of the calcite peak is slightly shifted in the ALD films, which can be due to imperfect sample alignment (Au or Si substrates were attached to standard XRD holders). Other structural effects are also possible, however, because of very low peak intensity and small film thickness, such analysis is not feasible.*

S7. Scanning Electron Microscopy (SEM)

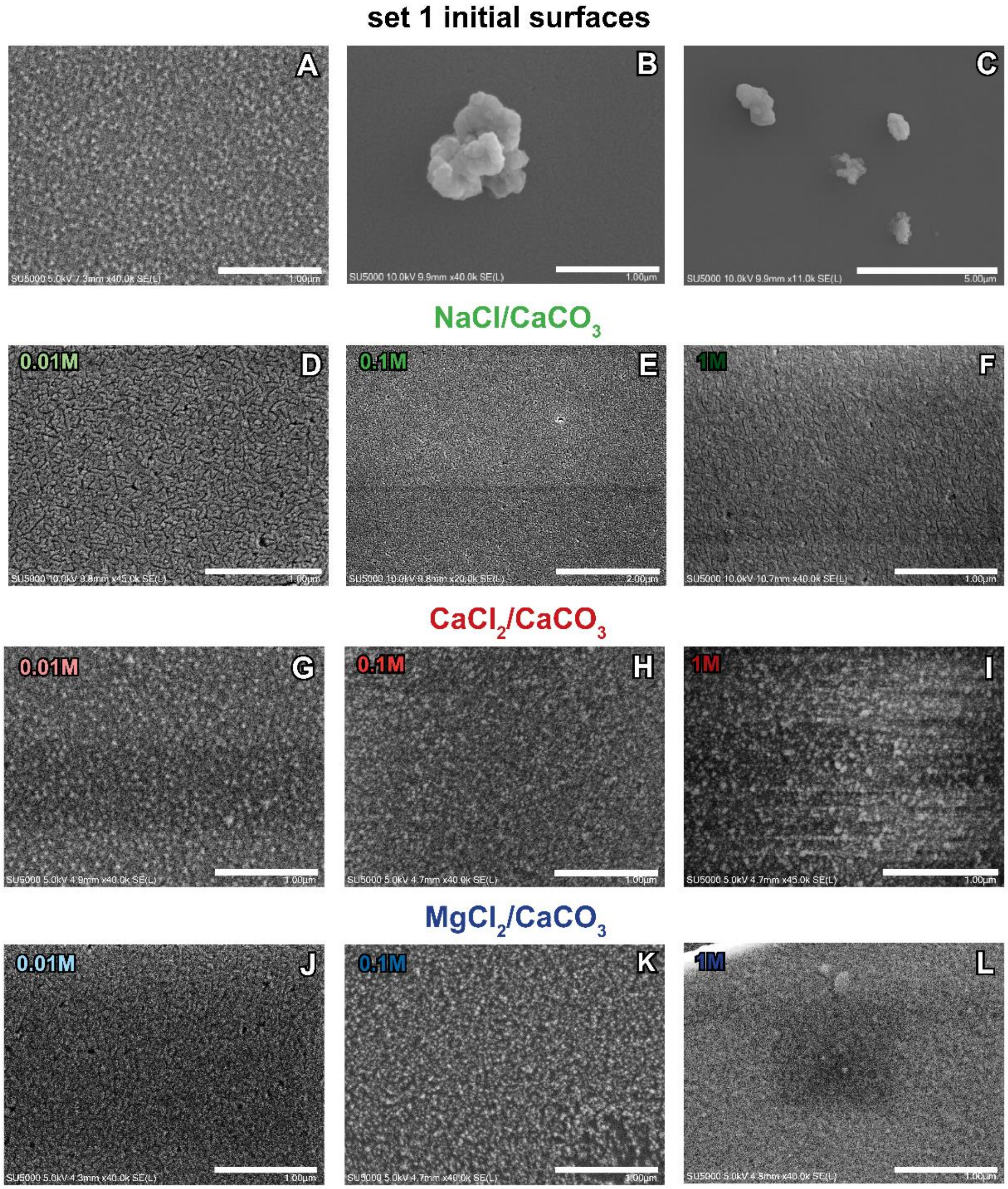

Figure S5. *SEM SE images of the initial (A-C) and final morphology (D-L) of the set 1 ALD calcite films. The D-L images show samples after the SFA experiments in NaCl, $CaCl_2$ and $MgCl_2$ solutions with ionic strength ranging from 0.01 to 1 M. Before the observations, samples were dried with N_2. Scale bars are 1 µm (C: 5 µm, E: 2 µm). Samples coated with Au. Visible $CaCl_2$ or $MgCl_2$ salt residue is visible on images H, I and K due to drying the wet samples after the SFA experiments with pressurized N_2.*

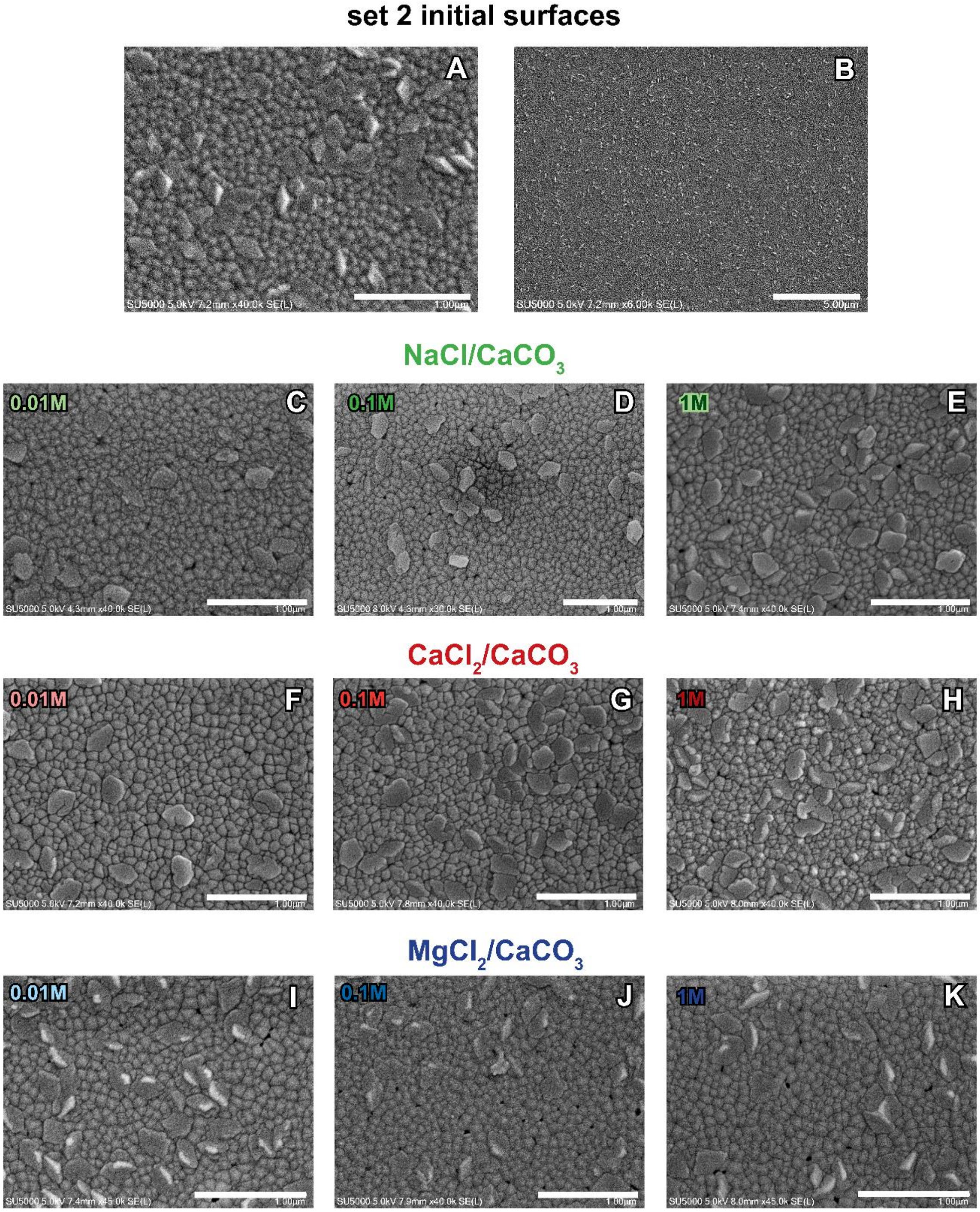

Figure S6. *SEM SE images of the initial (A-B) and final morphology (C-K) of the set 2 ALD calcite films. The C-K images show samples after the SFA experiments in NaCl, $CaCl_2$ and $MgCl_2$ solutions with ionic strength ranging from 0.01 to 1 M. Before the observations, samples were dried with N_2. Scale bars are 1 µm (B: 5 µm). Samples coated with Au.*

S8. Atomic Force Microscopy (AFM)

ALD films roughness characterization

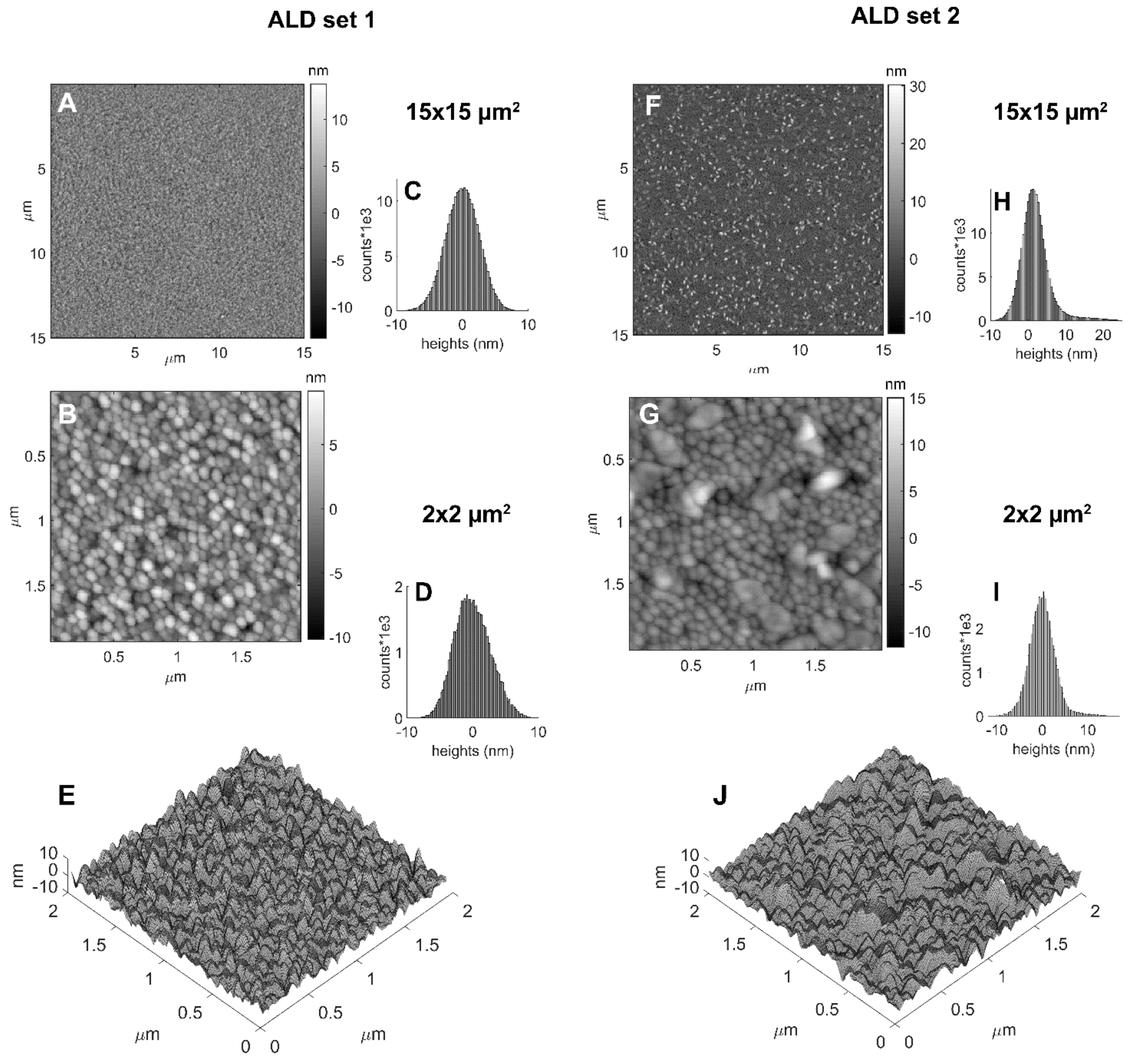

Figure S7. *AFM height maps (A, B, E, F, G, J) and histograms of surface heights (C, D, H, I) of the initial set 1 (A-E) and set 2 (F-J) ALD calcite surfaces for two scan sizes of 15x15 µm² (A, C, F, H) and 2x2 µm² (B, D, E, G, I, J). The images E and J show 3D height maps of the B, G height maps, respectively.*

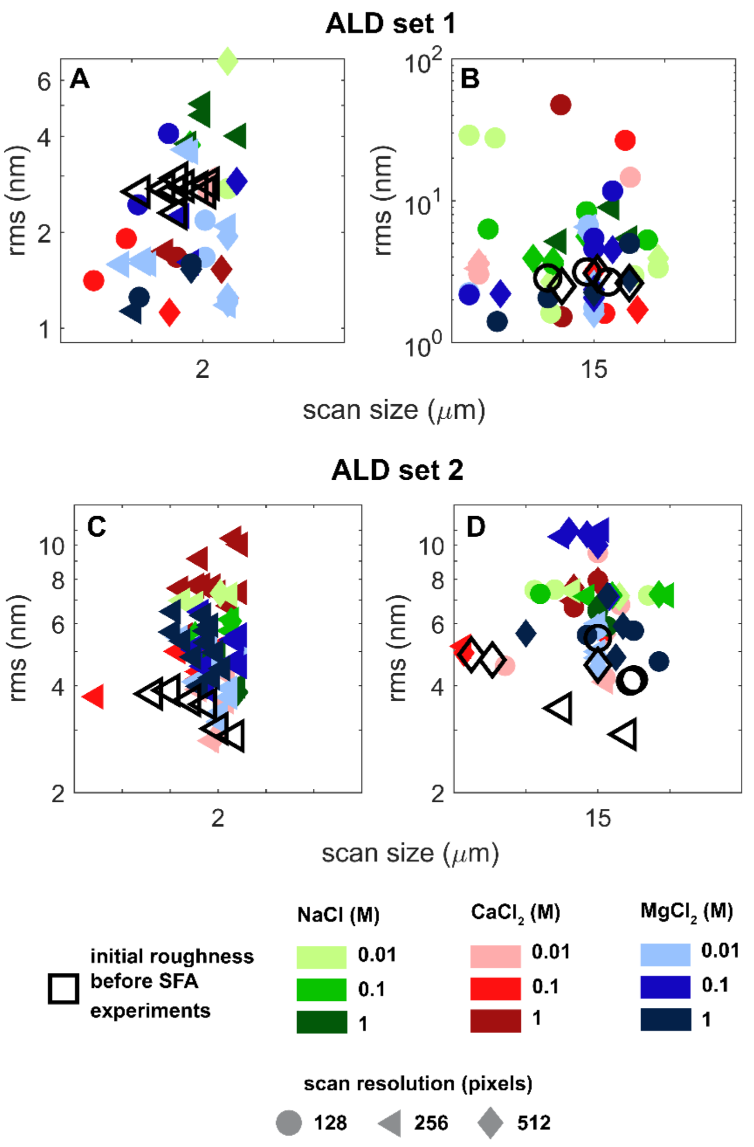

Figure S8. *AFM rms roughness parameters for the set 1 (A, B) and set 2 (C, D) ALD calcite surfaces for scan sizes of 15x15 µm² (B, D) and 2x2 µm² (A, C). Empty symbols correspond to rms values measured for the initial ALD surfaces before the SFA experiments. Colored symbols correspond to rms values measured for samples used in the SFA experiments in salt solutions with ionic strength (IS) varying from 0.01 to 1 M. For each sample we measured roughness in three random locations on the calcite surface, as it was not possible to locate where were the contacts used in the SFA measurements. Note a different y-scale in the B image. All salt solutions were presaturated with $CaCO_3$ as described in the Methods section.*

Equilibration of single calcite surfaces with solutions in the AFM

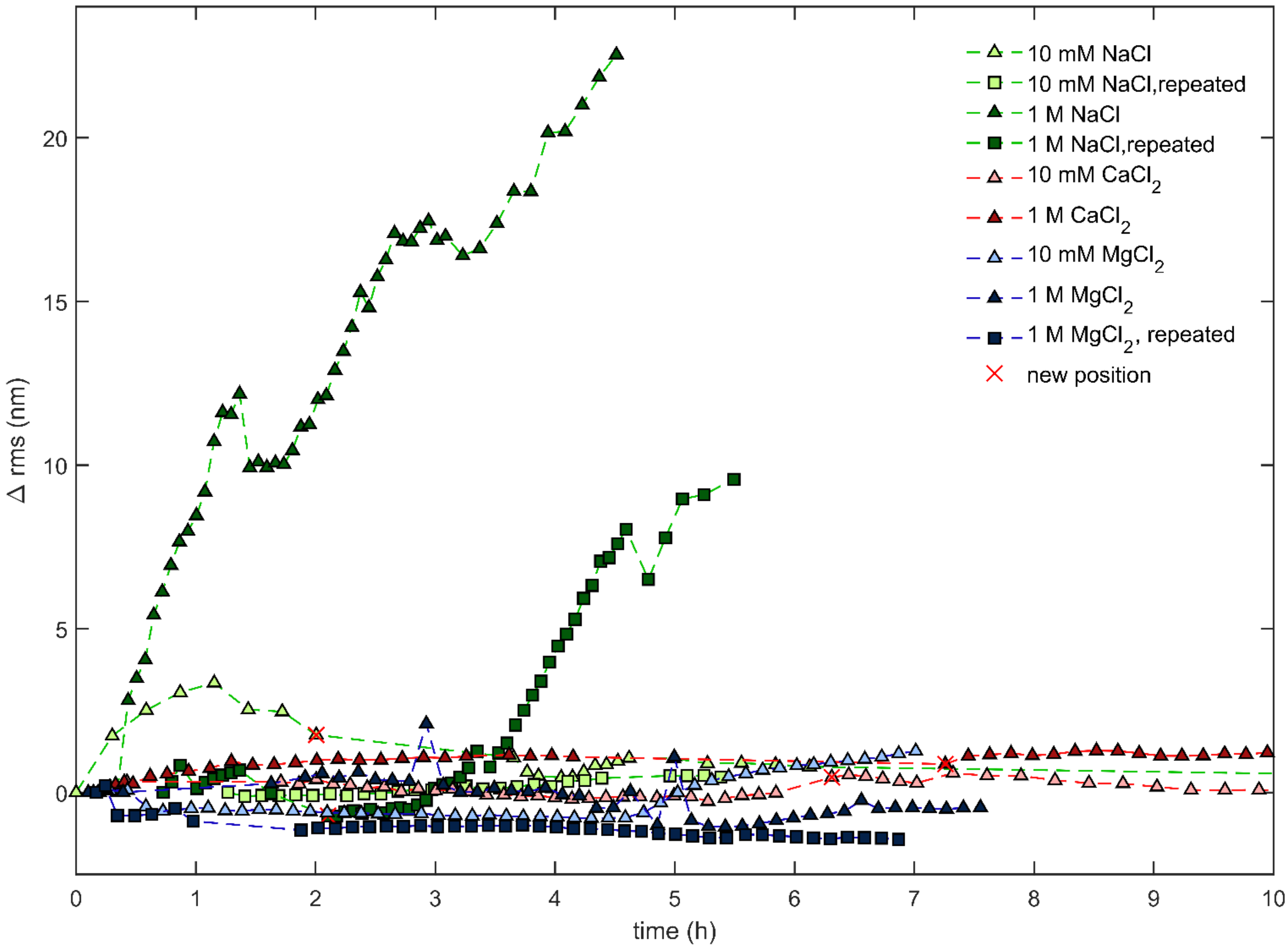

Figure S9. *AFM rms roughness evolution for the single, unconfined ALD calcite surfaces (set 3; scan size of 3x3 µm²) in NaCl, $CaCl_2$ and $MgCl_2$ salt solutions with ionic strengths of 0.01 or 1 M. All salt solutions were presaturated with $CaCO_3$ as described in the Methods section. The red x symbols mark changes in scanning position on a sample whenever the signal was lost due to a large instrumental drift. We observed major changes in surface roughness (see Figure S7) only for the experiments in 1 M IS NaCl/$CaCO_3$ solutions. The details of the measurements are given in the Methods section.*

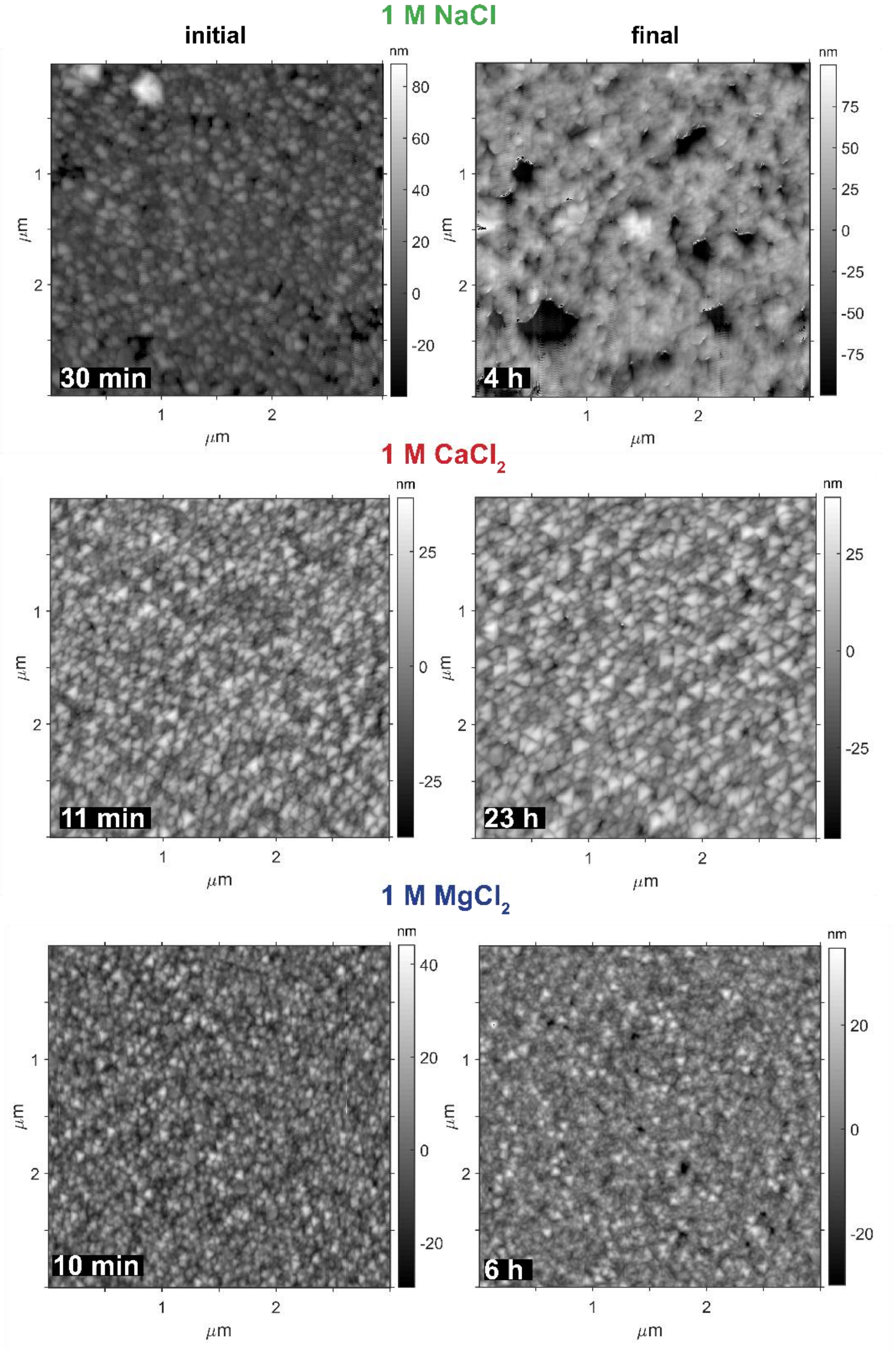

Figure S10. *AFM height maps for the set 3 ALD calcite surfaces (scan size of 3x3 μm^2) at different stages of the AFM roughness evolution measurements with single unconfined calcite surfaces (corresponding to Figure S6). We observed major changes in surface morphology only for the most concentrated 1 M NaCl/$CaCO_3$ solutions, for which μm-sized dissolution pits were developing everywhere on the surface. We observed no major changes in topography for experiments in other 0.01 and 1 M ionic strength solutions.*

S9. Modelling of Forces

Origin of the repulsive forces before the precipitation fronts – supplementary discussion

When modelling the contributions to the experimentally measured forces, we considered possible contributions of: Van der Waals (VdW) forces, hydration forces, roughness and electric double layer (EDL) forces. As explained below, we neglected the attractive VdW forces and repulsive hydration forces.

In general, DLVO theory predicts relatively strong Van der Waals (VdW) attractive forces (Hamaker constant of $1.44 \cdot 10^{-20}$ J for two calcite surfaces across water[10]) to act between calcite surfaces. This attraction is however weakened by hydration of hydrophilic calcite surface, which gives rise to structural repulsive hydration forces[11-13]. Therefore, in previous experiments, adhesive forces between two calcite surfaces have been measured only in high electrolyte concentrations (>0.1 M)[14] or at high pH (12)[15], and attributed to the collapse of the surface hydration layer and strengthening of ion-ion correlation forces[14], or EDL screening at low calcite zeta potentials[15]. Since we did not resolve any attraction nor adhesion, we did not include the VdW contribution.

Likewise, we neglected hydration, which is typically a nm-range, monotonically decaying repulsive force, generally described with decay lengths <2 nm for smooth surfaces[12]. Due to the unconventional Stern layer of calcite, where water molecules are directly adsorbed to the surface[16,17], both strong primary hydration (due to these adsorbed water layers) and secondary hydration related to surface-adsorbed cations are expected to act between calcite surfaces[12]. Hydration repulsion of a weaker magnitude has been reported in solutions with high electrolyte concentrations[11], a trend that roughly follows EDL interactions for calcite surfaces. It is likely that hydration contributes to the repulsive forces that we measured. However, since there are only semi-empirical expressions accessible to account for hydration repulsion, which do not include ion concentrations directly[12], and since hydration will be greatly affected by surface-specific ion binding, we did not include this interaction in our modelling. Additionally, as the hydration repulsion is a very short-range interaction, it cannot explain the very long-range repulsive force measured in some of our experiments (e.g. NaCl/$CaCO_3$ solutions Figure 2d). We expect that hydration repulsion can only act between the highest surface asperities on the rough calcite surfaces, when these asperities are placed in a direct contact at the smallest separations during the force-distance measurements. A more detailed discussion on hydration forces between rough calcite surfaces has been included in Dziadkowiec, et al. [1].

Electrical Double Layer (EDL) Forces Modelling

EDL forces were estimated for calcite surfaces using the three following equations, assuming calcite surface potential (ψ_0) of 5 or 30 mV (Figure 8).

a) Linear superposition approximation (LSA) method at constant potential (CP-LSA), adapted from Israelachvili [18] (see Figure 14.10, Chapter 14, page 317 therein):

$$EDL_{CP-LSA} = \kappa\sqrt{R^2}Ze^{-\kappa D}, \text{ where} \qquad \text{(Eq. S1)}$$

$$\kappa = \sqrt{\sum_i \frac{C_i e^2 z_i^2}{\varepsilon_0 \varepsilon kT}},$$

$$Z = 64\pi\varepsilon_0\varepsilon(kT/e)^2 tanh^2(\frac{ze\psi_0}{4kT}),$$

κ^{-1} is Debye length (m^{-1}), C_i is bulk concentration of each ion species i in the solution (M), z is ion valency, ε_0 is electrical permittivity of vacuum (F/m), ε is the water dielectric constant, k is the Boltzmann constant, T is temperature (K), R is the radius of the SFA cylindrical samples (m), and D is the distance between the surfaces (m). For mixed 2:1 $CaCl_2$ and $MgCl_2$ we assumed z = 2. For NaCl solutions we used z = 1.

b) Linearized Poisson-Boltzmann equation adapted from Diao and Espinosa-Marzal [11] and Trefalt, et al. [19] assuming a constant charge regulation parameter of calcite (p_c = 0.62), that has been experimentally determined by Diao and Espinosa-Marzal [11] in a calcite-silica system:

$$EDL_{CR} = 4\pi R\varepsilon\varepsilon_0\kappa\psi_0^2 \frac{e^{-\kappa D}+e^{-2\kappa D}(2p_c-1)}{1-(2p_c-1)^2 e^{-2\kappa D}} \qquad \text{(Eq. S2)}$$

c) simplified EDL force expression at low constant surface potential (<25 mV), suitable for mixed electrolytes, adapted from Israelachvili [18] (see Chapter 14, equation 14.56, page 318 therein):

$$EDL_{CP} = 4\pi R\varepsilon\varepsilon_0\kappa\psi_0^2 e^{-\kappa D} \qquad \text{(Eq. S3)}$$

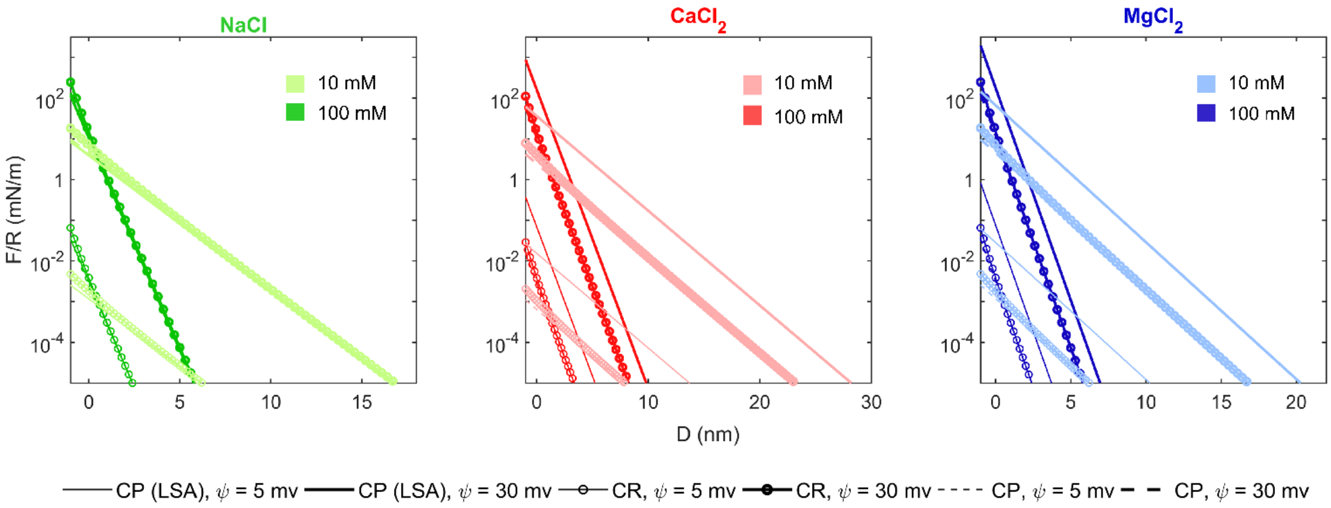

Figure S11. *Theoretical electrical double layer (EDL) force contributions estimated for two smooth calcite surfaces in 0.01 and 0.1 M IS electrolyte solutions. EDL forces were calculated using: a) linear superposition approximation (LSA) method at constant potential (CP (LSA); Eq. S1); b) linearized Poisson-Boltzmann equation at constant surface charge regulation using calcite surface charge regulation parameters adapted from Diao and Espinosa-Marzal [11] (CR, Eq. S2); c) simplified EDL force expression at constant surface potential, suitable for mixed electrolytes (CP, Eq. S3). EDL contributions were calculated assuming two values of calcite surface potential: 5 mV or 30 mV. Note that the EDL contributions calculated for NaCl solutions using all three expressions overlap, apart from the very small separations. Note that the EDL contributions calculated for $CaCl_2$ and $MgCl_2$ solutions using CR and CP expressions overlap, apart from the very small separations.*

S10. PhreeqC modelling

Calcite Dissolution Kinetics

Below we show an exemplary PhreeqC[20] input file, which was used to model calcite dissolution kinetics in NaCl, $CaCl_2$ and $MgCl_2$ salt solutions, with ionic strength ranging from 10 mM to 1 M (Figure 5B). The calculations were performed using the rate for calcite dissolution defined in $'llnl.dat'$ database, assuming open ($pCO_2 = 10^{-3.5}$) or closed systems ($pCO_2 = 10^{-6.2}$). The rate equation is based on the model for calcite dissolution proposed by Plummer, et al. [21]. It has to be noted that this simple PhreeqC model is based on empirically determined surface to solution volume ratio and is only meant to show the relative effect of electrolyte composition on calcite dissolution into salt solutions that are not saturated with respect to calcite.

```
# Calcite dissolution in 1M NaCl, system open for CO₂ (atmospheric pCO₂)

DATABASE llnl.dat

SOLUTION 1
    temp      25
    pH        7 charge
    pe        4
    redox     pe
    units     mmol/kgw
    density   1
    -water    1 # kg

EQUILIBRIUM_PHASES 1
    CO2(g)    -3.5    #atmospheric CO₂ pressure pCO₂ = 10^(-3.5) atm

REACTION 1
NaCl 1
0.01 in 1 steps

SAVE solution 1

END

USE solution 1
USE EQUILIBRIUM_PHASES 1

KINETICS 1 #using dissolution rate defined in llnl.dat database (Plummer, et al. 21)
       Calcite
       -m0 10e-3              # initial moles of calcite
       -parms 1.67e5 0.6
       # surface area of calcite (cm2/mol); exponent for surface change during dissolution
       -step 54000 in 1000    # total time 54000 seconds in 1000 steps
       -tol   1e-8            #integration tolerance

INCREMENTAL_REACTIONS true

USER_GRAPH
       -head time Ca pH
       -axis_titles "Time (h)" "Ca/mM" "pH"
       10 graph_x total_time/3600
       20 graph_y tot("Ca")*1e3
       30 graph_sy -la("H+")

END
```

Parameters of electrolyte solutions used in the SFA experiments

Below are two exemplary PhreeqC[20] input files, which were used to model pH and Ca^{2+} concentrations of the calcite-saturated NaCl/$CaCO_3$, $CaCl_2$/$CaCO_3$, and $MgCl_2$/$CaCO_3$, electrolyte solutions used in the SFA experiments (Figure 5C). We performed calculations in an open system with atmospheric partial pressure of CO_2 ($pCO_2 = 10^{-3.5}$ atm) and in a closed system with a reduced partial pressure of CO_2 ($pCO_2 = 10^{-6.2}$ atm).

```
# INPUT 1: 100 mM Nacl/CaCO3 solution saturated in an open system with atmospheric
# partial pressure of CO2

SOLUTION 1
    temp      25
    pH        7
    pe        4
    redox     pe
    units     mmol/kgw
    density   1
    Na        100   # concentration of NaCl - 100 mM
    Cl        100   # concentration of NaCl - 100 mM
    -water    1 # kg

EQUILIBRIUM_PHASES 1
    CO2(g)    -3.5  # partial pressure of CO2, 10^-3.5 atm
    Calcite   0 10  # SIcalcite = 0 (saturation index); excess initial
                          # amount of calcite set to 10 M



# INPUT 2: 100 mM Nacl/CaCO3 solution saturated in a closed system with partial
# pressure of CO2 ~ 0

SOLUTION 1
    temp      25
    pH        7
    pe        4
    redox     pe
    units     mmol/kgw
    density   1
    Na        100   # concentration of NaCl - 100 mM
    Cl        100   # concentration of NaCl - 100 mM
    -water    1 # kg

EQUILIBRIUM_PHASES 1
    CO2(g)    -6.2  # partial pressure of CO2, 10^-6.2 atm
    Calcite   0 10  # SIcalcite = 0 (saturation index); excess initial
                          # amount of calcite set to 10 M
```

S11. Surface Forces Apparatus (SFA) measurements

Calcite Thickness

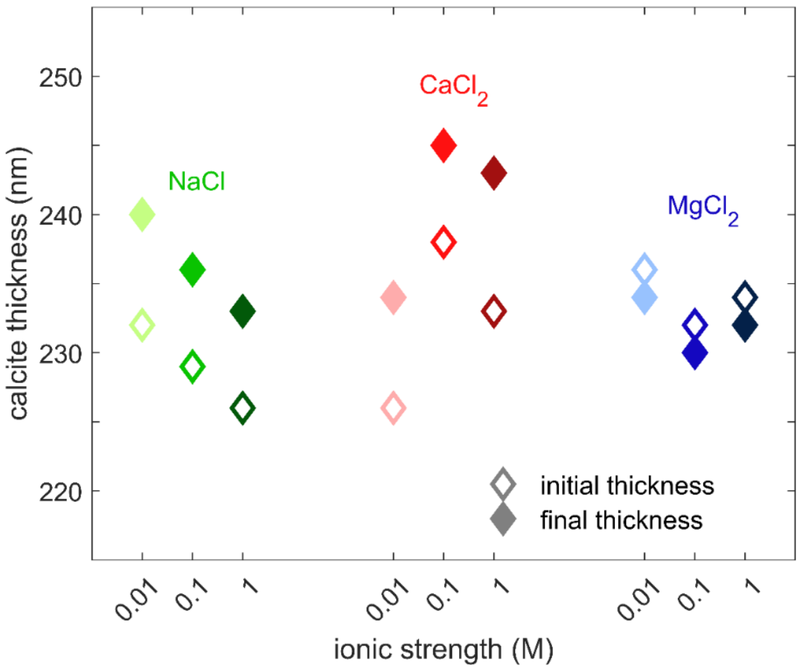

Figure S12. *Calcite thickness values estimated for the set 2 calcite surfaces in contact regions chosen for the SFA force measurements. The initial calcite thickness (empty symbols) was measured by placing the two calcite surfaces in contact at small applied loads using the SFA motor-driven micrometer. These small loads were usually sufficient for the set 2 surfaces to flatten in contact. The initial flattening indicated that separation between the surfaces was nm-ranged over the whole nominal contact areas*. The final thickness (filled symbols) was estimated at the end of experiments. Because the precipitate was present between the surfaces, very high loads had to be applied in order to place the surfaces in flattened contacts. We used a manual SFA micrometer control to achieve these high loads. The very small difference in the initial and final calcite thicknesses for experiments in NaCl and $CaCl_2$ solutions indicates that most of the precipitate was squeezed out from between the surfaces at high applied loads. For the set 2 experiments in $MgCl_2$ solutions, for which we did not observe PFs, there was a decrease in calcite thickness in the contact region.*

*Because of the nm-scale roughness of our ALD surfaces, only the highest asperities were in a direct contact, and separations varied across the large nominal contact areas (~100 µm in radius). Therefore, the plotted calcite thicknesses are only average values across the whole nominal contact areas, related to the distribution of the highest asperities. We also measured calcite thickness in one or two additional contact positions for each sample at the beginning of the experiments (not plotted here), and we obtained comparable calcite thickness values. That shows that the roughness and thickness of the set 2 calcite surfaces was homogenous over large areas of ALD films.

Details of the SFA measurements

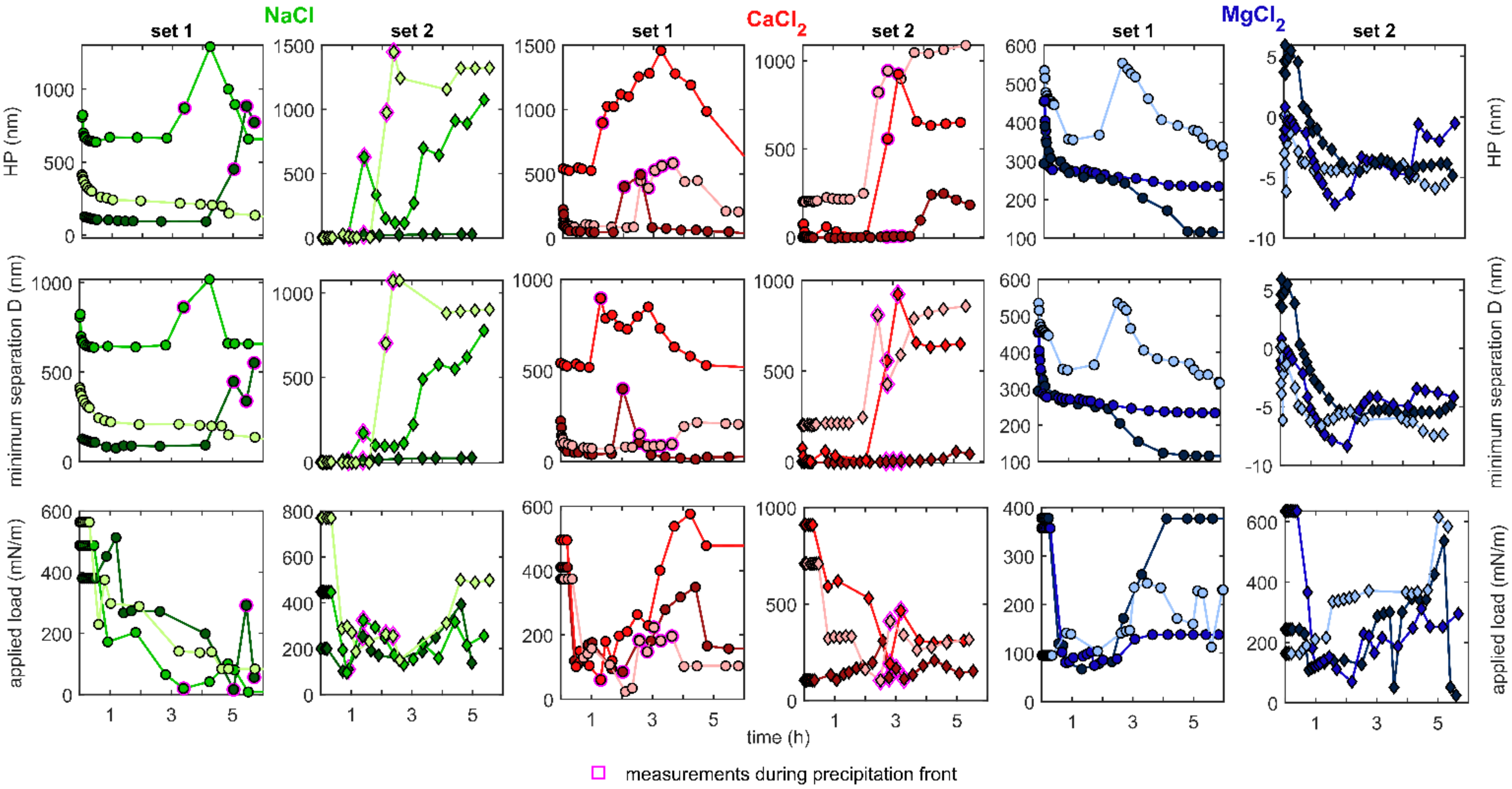

Figure S13. *Details of the SFA data shown in Figure 4. The middle panel shows minimum separation D at the maximum applied load as a function of elapsed time. The bottom panel shows maximum applied load as a function of elapsed time. Hardwall position (HP; top row) shows the separation between the surfaces at the applied load value common to all measurements for each experiment (the experimental points for each experiment are connected with solid lines; as in Figure 4). The colors correspond to ionic strength and composition of the used salt solutions that are consistent throughout the manuscript (e.g. Figure S5).*

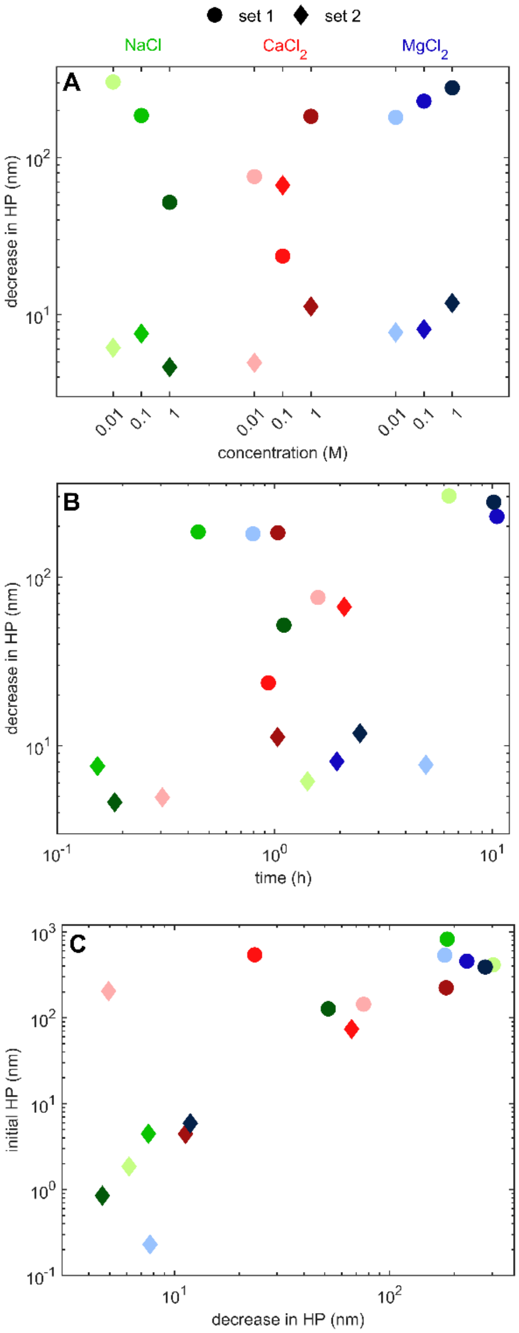

Figure S14. *Details of the SFA measurements for the set 1 (○) and set 2 (◊) surfaces (corresponding to the data shown in Figure 4). Colors indicate composition and ionic strength of the salt solutions used in the experiments (subplot A). A) Dependence of the decrease in HP measured before the PFs in the contact region used in the SFA experiments (or before the first progressive increase in HP for experiments in $MgCl_2$ solutions) on the ionic strength and composition of the used salt solutions; B) Dependence of the decrease in HP measured before the PFs in the contact region on the elapsed time; C) Dependence of the initial HP (first experimental point shown Figure 4 (top row) for each experiment on the decrease in HP measured before the PFs in the used contact region.*

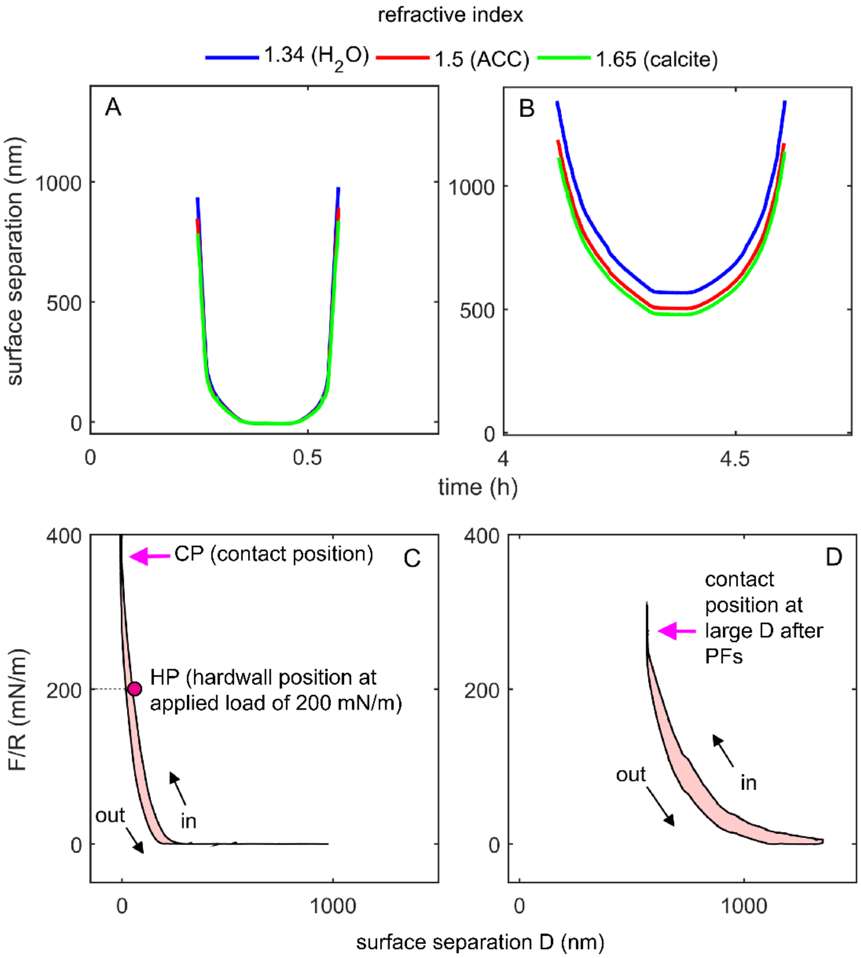

Figure S15. *Details of the SFA measurements showing effective surface separations modelled in Reflcalc (see the Methods section and* *Dziadkowiec, et al.* [1]*) using different values of the refractive index (n) for the solution confined between two calcite surfaces. SFA data from the set 2 experiment in 0.1 M IS $CaCl_2$ solution (see Figure 4). A) Surface separation as a function of time for one representative loading-unloading cycle, measured before the PF event. At the beginning of the experiment it was possible to reach the initial CP. There was only a little difference between the surface separations calculated using the different n values; B) Surface separation as a function of time for one representative loading-unloading cycle, measured after the PF event. At large surface separations, there was a substantial difference between the separations calculated using the three n values. Even if the highest n of calcite was used, the estimated surface separation was still large; C) Force-distance curve before PF, corresponding to the data in the subplot A. Locations of contact position (CP, separation at which distance between the surfaces no longer decreases despite continued loading) and hardwall position (HP, separation measured at a given applied load value) are indicated; D) Force-distance curve after PF, corresponding to the data in the subplot B. A contact position at very large separations is indicated, where the separation between the surfaces does not decrease further despite the continued loading.*

Note that for water and calcite we used the tabulated values of n (adapted from *Hale and Querry* [22] *and* *Ghosh* [23]*, respectively). The values for birefringent calcite were used as an average value for ordinary*

and extraordinary rays at a given wavelength, whereas for ACC we used a constant value of 1.5 due to lack of detailed n parameters for ACC[24]*. The legend shows values of n at wavelength of 600 nm for water and calcite (ordinary n).*

SFA experiment in monoethylene glycol

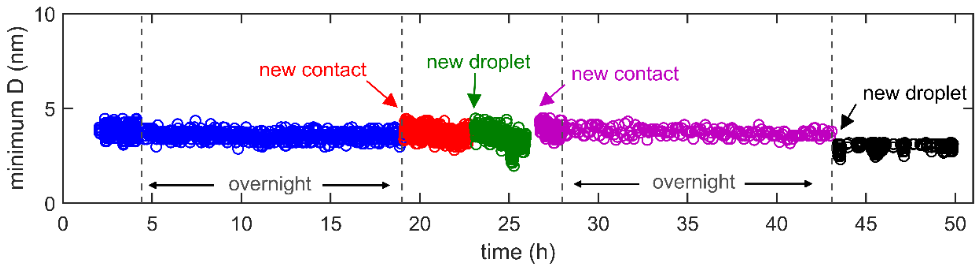

Figure S16. *Minimum separation between two calcite surfaces plotted as a function of elapsed time, measured in the SFA experiment in monoethylene glycol (MEG; ethane-1,2-diol; Merck, reagent grade, 99.5% pure). Data correspond to minimum separations measured during the consecutive force-distance runs or to periods of time when the surfaces were kept in contact under the constant applied load ('overnight'). Only a droplet of MEG (~ 2 ml) was injected between the surfaces. At the beginning of the experiment, MEG solution was exchanged multiple times to ensure a complete surface wetting. The arrows indicate when a contact position between the two calcite samples was changed or when MEG droplet was replaced with fresh MEG solution. MEG was not presaturated with calcite. Please note that we did not observe any precipitation events for 3-days experiments in MEG.*

Crystals growing in SFA contact regions at high applied loads

We attempted to locate the crystals that grew in SFA experiments in a contact region after precipitation front events when pushing two calcite surfaces against each other manually at very high loads (see Figure 4, Supplementary Movies 16 and 18). We imaged calcite samples after the SFA experiment with Scanning Electron Microscope (SEM; using samples from the SFA experiment shown in section S4 in 100 mM NaCl/$CaCO_3$ solution). In order not to remove the rather loose, larger particles that grew in the contact region we did not rinse the samples and we let the NaCl salt solution evaporate on the samples. Because of that, the samples were covered in NaCl. Although it was not possible to unambiguously locate the contact region used in the SFA experiment, we found one area that could correspond to the contact region with µm-sized $CaCO_3$ particles on the surface (Figure S17). In this area, we could identify some larger particles that were $CaCO_3$ crystals, but it was not possible to confidently say that these particles were the ones observed during the SFA experiment. The µm-sized $CaCO_3$ aggregates were partially coated with evaporated salt and some of them were located on top of the NaCl salt (Figure S17C). This may indicate that they were only loosely attached to the surface, similarly to the particles observed in the SFA experiments.

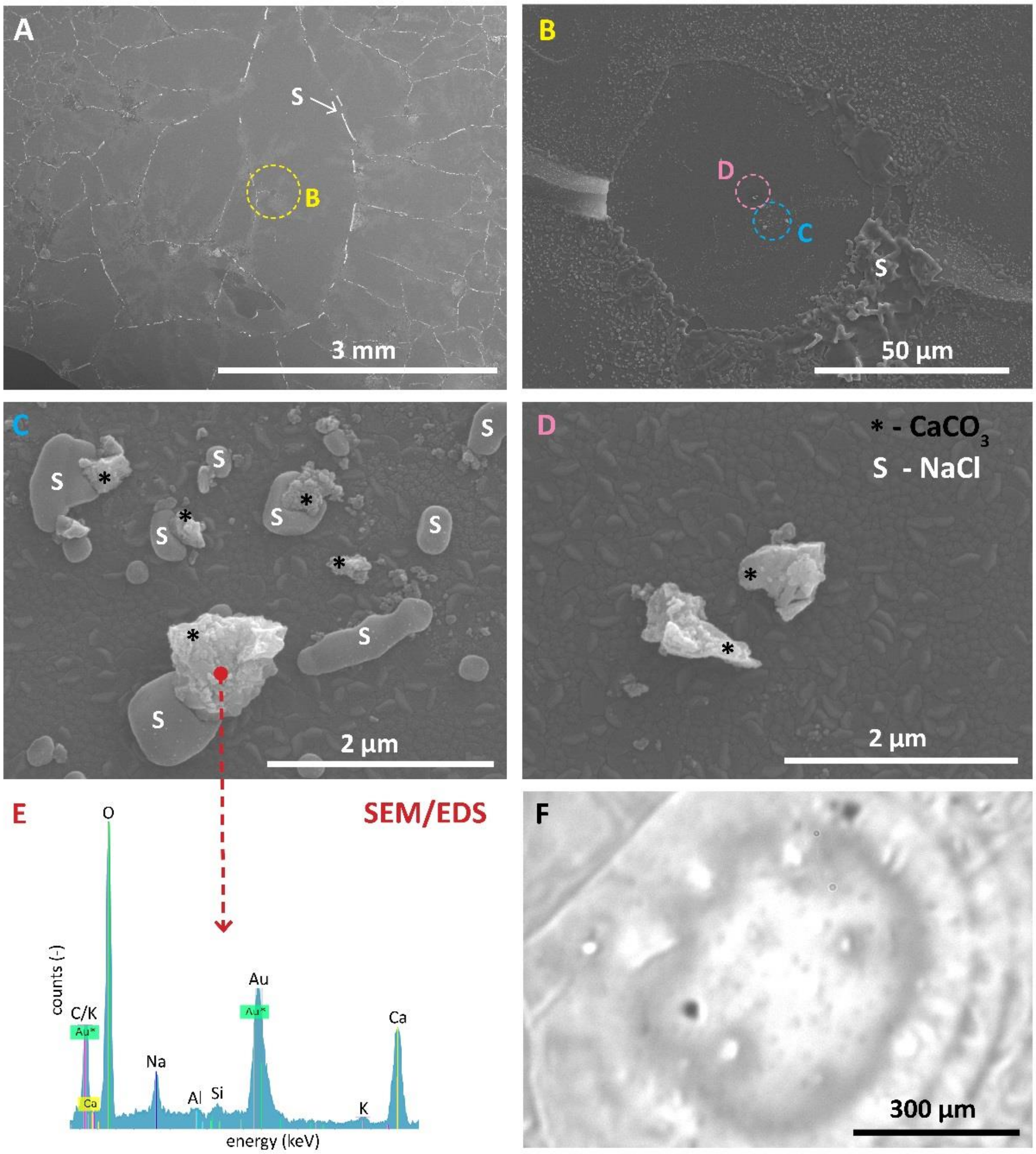

Figure S17: *A-D) Scanning Electron Microscopy (SEM) images of the ALD calcite film sample showing a region possibly used as a contact region in the SFA experiment (NaCl 100 mM/$CaCO_3$, set 2, see section S4). In this experiment, we pushed two calcite surfaces against each other at very high loads and we observed the formation of large crystals (F; supplementary movie 18). Large polycrystalline aggregates (*) were identified in the center of the possible contact region with SEM (C-D). SEM/EDS (energy dispersive X-ray spectroscopy) analysis (E) indicated that these aggregates were $CaCO_3$ (elements K, Al, and Si come from the underlying mica substrates and Au is due to the gold coating on the sample). Smoother crystals (S) were NaCl evaporates (the sample was not rinsed after the experiment in order not to lose the large crystals that grew in the contact region (F). The optical in situ image of the contact region during the SFA experiment (F) showed larger crystals that the ones we observed with SEM. However, as these crystals were not attached to the underlying calcite substrates, it is possible that the largest crystals were rinsed off from the surface when we took the calcite surfaces out of the SFA chamber at the end of the experiment. Cracked surface of the sample (A) is due to drying; we never observe cracking during experiments.*

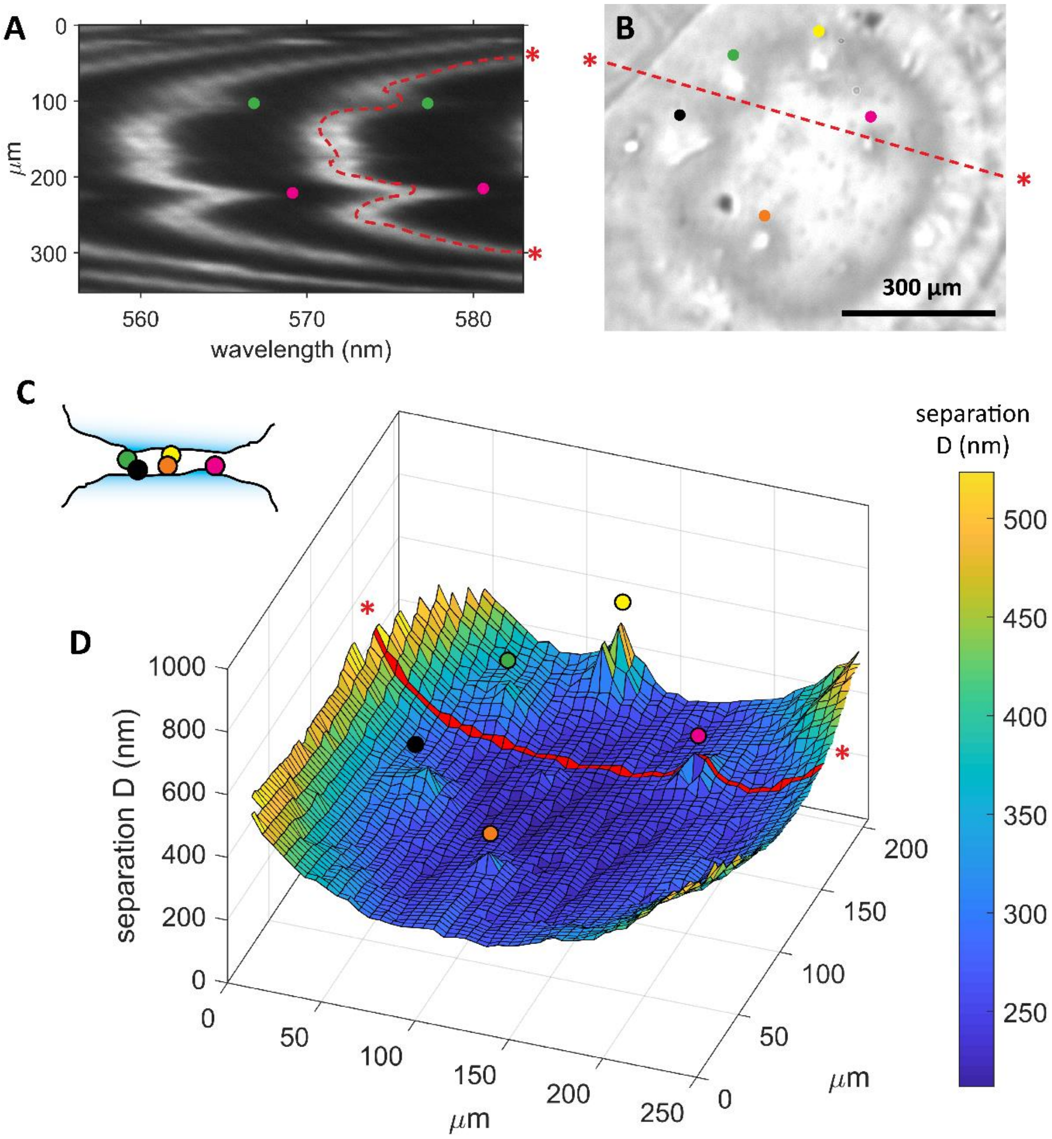

Figure S18: *Contact region used to measure forces between two calcite surfaces in the SFA experiment, after pushing the surfaces against each other manually at very high loads (see Supplementary Movie M18; SFA experiment in 100 mM NaCl/$CaCO_3$ solution saturated in open system; section S4). The surfaces were pushed against each other after the precipitation front event. The precipitate confined between the surfaces underwent ripening, leading to the formation of µm-sized crystals. The crystals are marked on each panel with correspondingly colored dots; A) Exemplary FECO fringes showing separation between the two calcite surfaces along the red line marked with an asterisk (*). Large irregularities in the FECO fringes (marked with green and pink dots) correspond to big crystals trapped between two calcite surfaces; B) In situ SFA optical micrograph of the contact region with µm-sized crystals visible on the surface. Newton rings visible on the surface connect the regions of the same surface separation; C) Sketch showing crystals trapped between calcite surfaces; D) Surface separation (D) map of the contact region reconstructed from FECO fringes when scanning over the whole contact area. The map indicates that the crystals trapped between calcite surfaces were not thicker than 500 nm.*

References

1 Dziadkowiec, J., Javadi, S., Bratvold, J. E., Nilsen, O. & Røyne, A. Surface Forces Apparatus Measurements of Interactions between Rough and Reactive Calcite Surfaces. *Langmuir : the ACS journal of surfaces and colloids* **34**, 7248-7263, doi:10.1021/acs.langmuir.8b00797 (2018).

2 Nilsen, O., Fjellvag, H. & Kjekshus, A. Growth of calcium carbonate by the atomic layer chemical vapour deposition technique. *Thin Solid Films* **450**, 240-247, doi:10.1016/j.tsf.2003.10.152 (2004).

3 Benjamin, M. M. *Water chemistry*. (Waveland Press, 2014).

4 Røyne, A., Bisschop, J. & Dysthe, D. K. Experimental investigation of surface energy and subcritical crack growth in calcite. *Journal of Geophysical Research: Solid Earth* **116** (2011).

5 Wolf, G. & Günther, C. Thermophysical investigations of the polymorphous phases of calcium carbonate. *J Therm Anal Calorim* **65**, 687-698 (2001).

6 Bischoff, J. L., Fitzpatrick, J. A. & Rosenbauer, R. J. The solubility and stabilization of ikaite (CaCO3· 6H2O) from 0 to 25 C: Environmental and paleoclimatic implications for thinolite tufa. *The Journal of Geology* **101**, 21-33 (1993).

7 Plummer, L. N. & Busenberg, E. The solubilities of calcite, aragonite and vaterite in CO2-H2O solutions between 0 and 90 C, and an evaluation of the aqueous model for the system $CaCO_3$-CO_2-H_2O. *Geochimica et cosmochimica acta* **46**, 1011-1040 (1982).

8 Hull, H. & Turnbull, A. A thermochemical study of monohydrocalcite. *Geochimica et Cosmochimica Acta* **37**, 685-694 (1973).

9 Nilsen, O., H., F. & Kjekshus, A. Growth of calcium carbonate by the atomic layer chemical vapour deposition technique. *Thin Solid Films* **450**, 240-247 (2004).

10 Bergström, L. Hamaker constants of inorganic materials. *Adv Colloid Interfac* **70**, 125-169 (1997).

11 Diao, Y. & Espinosa-Marzal, R. M. Molecular insight into the nanoconfined calcite–solution interface. *Proceedings of the National Academy of Sciences* **113**, 12047-12052 (2016).

12 Donaldson Jr, S. H. *et al.* Developing a general interaction potential for hydrophobic and hydrophilic interactions. *Langmuir : the ACS journal of surfaces and colloids* **31**, 2051-2064 (2014).

13 Røyne, A., Dalby, K. N. & Hassenkam, T. Repulsive hydration forces between calcite surfaces and their effect on the brittle strength of calcite-bearing rocks. *Geophys Res Lett* **42**, 4786-4794, doi:10.1002/2015gl064365 (2015).

14 Javadi, S., & Røyne, A. (2018). Adhesive forces between two cleaved calcite surfaces in NaCl solutions: The importance of ionic strength and normal loading. Journal of colloid and interface science, 532, 605-613.

15 Pourchet, S., Pochard, I., Brunel, F. & Perrey, D. Chemistry of the calcite/water interface: influence of sulfate ions and consequences in terms of cohesion forces. *Cement Concrete Res* **52**, 22-30 (2013).

16 Stipp, S. Toward a conceptual model of the calcite surface: hydration, hydrolysis, and surface potential. *Geochimica et Cosmochimica Acta* **63**, 3121-3131 (1999).

17 Geissbühler, P. *et al.* Three-dimensional structure of the calcite–water interface by surface X-ray scattering. *Surf Sci* **573**, 191-203 (2004).

18 Israelachvili, J. N. *Intermolecular and surface forces*. (Academic press, 2015).

19 Trefalt, G., Ruiz-Cabello, F. J. M. & Borkovec, M. Interaction forces, heteroaggregation, and deposition involving charged colloidal particles. *The Journal of Physical Chemistry B* **118**, 6346-6355 (2014).

20 Parkhurst, D. L. & Appelo, C. Description of input and examples for PHREEQC version 3: a computer program for speciation, batch-reaction, one-dimensional transport, and inverse geochemical calculations. Report No. 2328-7055, (US Geological Survey, 2013).

21 Plummer, L., Wigley, T. & Parkhurst, D. The kinetics of calcite dissolution in CO_2-water systems at 5 degrees to 60 degrees C and 0.0 to 1.0 atm CO_2. *Am J Sci* **278**, 179-216 (1978).

22 Hale, G. M. & Querry, M. R. Optical constants of water in the 200-nm to 200-μm wavelength region. *Applied Optics* **12**, 555-563 (1973).

23 Ghosh, G. Dispersion-equation coefficients for the refractive index and birefringence of calcite and quartz crystals. *Opt Commun* **163**, 95-102 (1999).

24 Merten, H. L. & Bachman, G. L. (Google Patents, 1980).
